# Mechanisms underlying the health effects of desert sand dust

**DOI:** 10.1016/j.envint.2021.106790

**Published:** 2021-12

**Authors:** Julia C. Fussell, Frank J. Kelly

**Affiliations:** National Institute for Health Research Health Protection Research Unit in Environmental Exposures and Health, School of Public Health, Sir Michael Uren Building, Imperial College London, White City Campus, 80-92 Wood Lane, London W12 0BZ, United Kingdom

**Keywords:** Allergic respiratory disease, Asian sand dust, Cardiovascular disease, Desert dust, Inflammation, Microbial materials, Oxidative stress, Toxicology

## Abstract

Desertification and climate change indicate a future expansion of the global area of dry land and an increase in the risk of drought. Humans may therefore be at an ever-increasing risk of frequent exposure to, and resultant adverse health effects of desert sand dust. This review appraises a total of 52 experimental studies that have sought to identify mechanisms and intermediate endpoints underlying epidemiological evidence of an impact of desert dust on cardiovascular and respiratory health. Toxicological studies, in main using doses that reflect or at least approach real world exposures during a dust event, have demonstrated that virgin sand dust particles and dust storm particles sampled at remote locations away from the source induce inflammatory lung injury and aggravate allergen-induced nasal and pulmonary eosinophilia. Effects are orchestrated by cytokines, chemokines and antigen-specific immunoglobulin potentially via toll-like receptor/myeloid differentiation factor signaling pathways. Findings suggest that in addition to involvement of adhered chemical and biological pollutants, mineralogical components may also be implicated in the pathogenesis of human respiratory disorders during a dust event. Whilst comparisons with urban particulate matter less than 2.5 μm in diameter (PM_2.5_) suggest that allergic inflammatory responses are greater for microbial element-rich dust- PM_2.5_, aerosols generated during dust events appear to have a lower oxidative potential compared to combustion-generated PM_2.5_ sampled during non-dust periods. *In vitro* findings suggest that the significant amounts of suspended desert dust during storm periods may provide a platform to intermix with chemicals on its surfaces, thereby increasing the bioreactivity of PM_2.5_ during dust storm episodes, and that mineral dust surface reactions are an unrecognized source of toxic organic chemicals in the atmosphere, enhancing toxicity of aerosols in urban environments. In summary, the experimental research on desert dust on respiratory endpoints go some way in clarifying the mechanistic effects of atmospheric desert dust on the upper and lower human respiratory system. In doing so, they provide support for biological plausibility of epidemiological associations between this particulate air pollutant and events including exacerbation of asthma, hospitalization for respiratory infections and seasonal allergic rhinitis.

## Highlights

• 52 studies investigating mechanisms behind impacts of desert dust on health are reviewed.
• Desert dust may be a risk factor for inflammatory and allergic lung diseases.
• Adhered chemicals, biological and mineralogical components are candidate activators.
• Desert dust surface reactions may enhance toxicity of aerosols in urban environments.

## 1 Introduction

Desert dust storms have the potential to elicit adverse health effects on a global scale owing to the transportation of dust particles over long distances (Esmaeil et al. 2014). In certain parts of the world, though not all, the frequency and scale of dust storms have increased in response to land use and climatic changes (Goudie 2014). Current environmental fluctuations such as desertification and climate change indicate a future expansion of the global area of dry land (Huang et al. 2015) and an increase in the risk of drought (Dai 2012). Humans would therefore appear to be at an ever-increasing risk of frequent exposure to, and any adverse health effects of desert sand dust.

Whilst epidemiological studies suggest that such effects encompass several indices of ill health including daily mortality and cardiorespiratory diseases (Kanatani et al., 2010, Stafoggia et al., 2016, Ueda et al., 2012, Watanabe et al., 2011), reviews have reported inconclusive results (Karanasiou et al., 2012, Zhang et al., 2016). To address this, a recent systematic review and meta-analysis has been undertaken, accounting for the relevant dust patterns from source areas and emissions (Tobias et al., 2019a). This effort reported an increased risk of cardiovascular mortality (1.6%, 95% CI 0.0, 3.1) and respiratory morbidity (6.8%, 95% CI: 1.8, 11.9), but still concluded that the evidence is inconsistent when accounting for sources of particulate matter (PM) in different geographical areas (Tobias et al., 2019b).

To further assess certainty in the human evidence, biological plausibility can be sought, by identifying a mechanism, or set of mechanisms, by which desert dust particles could cause adverse health outcomes. In the absence, to our knowledge of such an exercise, we have conducted a qualitative appraisal of experimental studies that have sought to identify mechanisms and intermediate endpoints underlying epidemiological evidence of an impact of desert dust on cardiovascular and respiratory health.

## 2 Methods

**Fig. 1:**
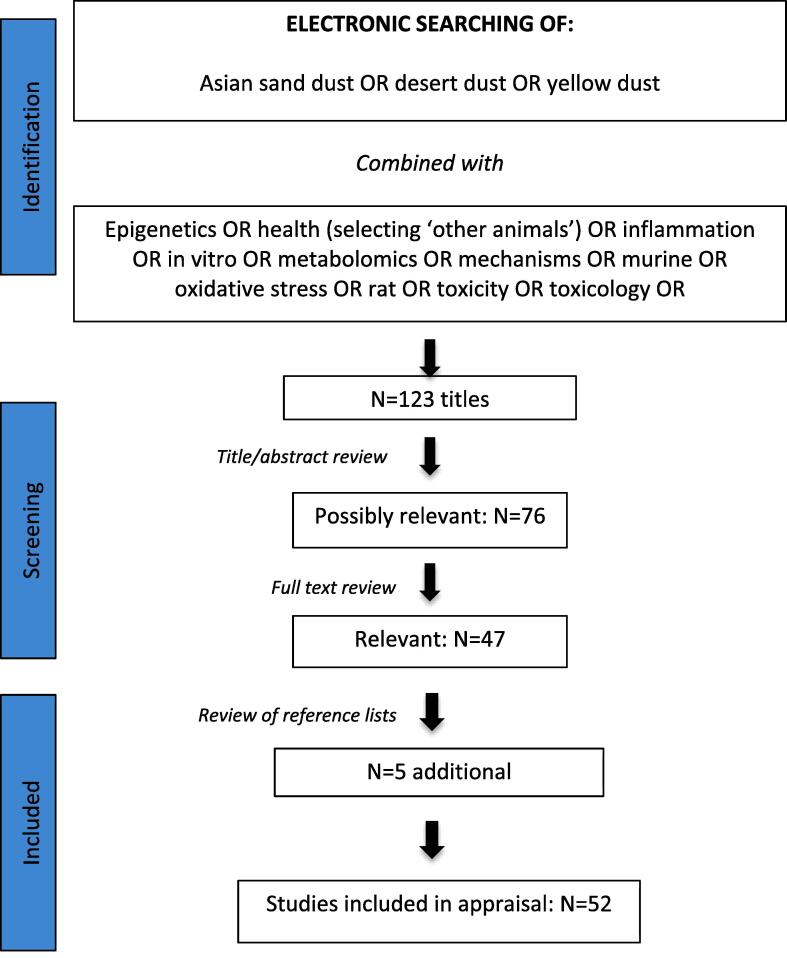
Study identification and selection.

The studies appraised in this review were identified using a PubMed search for all articles published before November 2019 using all permutations of key words listed in Fig. 1. Oxidative stress and inflammation were included as key words as scientific consensus continues to grow that the capacity of inhaled PM to elicit these pathways both within the lung and systemically, is a central mechanism leading to respiratory and cardiovascular ill health observed in exposed populations (Kelly and Fussell, 2017). For example, particulate air pollution can induce airway inflammation (Dales et al. 2008), a feature of asthma, as well as oxidative stress (Liu et al., 2009, Patel et al., 2013), a feature of the severe disease. Experimental work has also provided strong evidence that oxidative pathways are an important cause and consequence of PM-mediated cardiovascular events (Miller, 2020).

The titles and abstracts of 123 original research and review articles were screened. Those that were not relevant to the focus of this review were discarded, leaving 76 articles that were then appraised in detail. Of these, 47 studies were deemed relevant to which an additional 5 papers were identified from hand searching reference lists of primary search results. Only publications in English were considered.

## 3 Results

Mechanistic studies have focused on virgin sand dust particles (ie collected from surface soils at the source) plus dust storm particles. The latter encompass ‘wind blown’ or ‘ambient’ sand dust sampled at a remote location away from the source. Comparisons of the toxicity between these different dust types help to determine contributions from the dust particles themselves (sand dust composed mainly of silicon, aluminum, calcium and iron) as well as other possible responsible factors such as the various biological and chemical materials that are travel with and/or are adhere to sand dust during long-range transportation. The latter include soil derived microorganisms, as well as toxic chemicals such as polycyclic aromatic hydrocarbons (PAH) from industrial pollution (Tamamura et al. 2007). The most studied microorganisms in this context are those known to induce or aggravate a variety of respiratory diseases, namely β-glucan and lipopolysaccharide (LPS), the major structural components of Gram-negative bacteria (Nikaido 1969) and fungi (Hearn and Sietsma 1994) respectively. Dust derived from alkaline soil also captures acid gases such as sulphur and nitrogen oxides that are produced from fossil fuel combustion in industrialised areas. Once adsorbed onto particles of sand, sulphur dioxide (SO_2_) and nitrogen dioxide (NO_2_) subsequently form sulphates (SO_4_^2−^) or nitrates respectively (H. He et al., 2014, Karydis et al., 2016, Yu et al., 2020).

The vast majority of the research undertaken has focused on Asian sand dust (ASD; dust originates from wind erosion in arid and semiarid areas of middle and northwestern China), whilst other geographical areas from which desert dust has been studied include Arizona, Nevada, Sahara and the Middle East. By far the most commonly studied organ system within the animal toxicological literature is the lung, in which general cytotoxicity/damage, (allergic) inflammation, infection and oxidative stress has been examined (Table S1). A proportion of these *in vivo* studies have an accompanying *in vitro* component and in this case, the two components are summarized together in both the commentary below and in Table S1. The stand alone *in vitro* studies have focused on cytotoxicity and respiratory endpoints (Table S2).

### 3.1 Animal studies (see Table S1)

#### 3.1.1 Dosages employed

Owing to the exceptionally high ambient concentrations of PM that are experienced during dust storms, with quoted figures that can range from 100 μg/m^3^ (Lei et al., 2004) to 6000 μg/m^3^ (Ichinose et al., 2005), many of the studies summarized below have adopted doses that reflect or at least approach real world exposures. Efforts have also been made in extrapolating intratracheal (IT) doses employed to likely concentrations inhaled during a dust event. As an example, 300 and 600 μg ASD have been instilled into guinea pigs that at 14 weeks have a body weight of about 512 g, a tidal volume of approximately 1.8 ml and breathing rate of about 94 breaths per min (Ichinose et al. 2009). The calculation was made that animals inhale 0.24 m^3^ air per day and therefore 24 μg suspended PM (SPM) per day (or 170 μg/week) using the Japanese SPM national air quality standard of 100 μg/m^3^ or about 115 μg per week assuming a 68% deposition rate for a 6 μm diameter particle (James et al. 1994). The 300 and 600 μg particle instillation doses employed were therefore calculated to be approximately 2.6 and 5.2 times higher than the SPM standard respectively. However since SPM concentrations are 2–6 times higher than the national air quality standard when Asian dust storm events occur (Hayasaki et al., 2007, Mori, 2003) the instillation doses of particles were considered close to the concentrations of a real desert sand dust world environment. Similar calculations have been made for rats, based on their tidal air volume and breathing rate at a given weight, and from these a dose of 400 μg is considered to be of similar magnitude to the volume of ASD particles that could have been inhaled on seven consecutive days during the ASD season in Japan (Shimada et al., 2015).

#### 3.1.2 Lung damage / inflammation

##### 3.1.2.1 Asian sand dust

Early and highly quoted work suggesting ASD can cause pulmonary inflammation and lung damage includes that of Lei et al. who studied effects of ambient ASD (AASD; also referred to as Asian dust storm particles [ADSP]) on peripheral blood and bronchoalveolar lavage fluid (BALF) from male pulmonary hypertensive rats (Lei et al. 2004). Animals were exposed employing a nose-only inhalation system to concentrated ambient particles (CAPs; 315.6 μg/m^3^ for 6 h or 684.5 μg/m^3^ for 4.5 h). This took place during the peak of a dust storm when concentrations of particulate matter less than 10 μm in diameter (PM_10_) monitored near Chung-Li, Taiwan, were greater than 100 μg/m^3^ and concentrations of silica (SiO_2_) were 10-fold higher than periods without dust storms. Thirty-six hours after exposure, numbers of white blood cells in the peripheral blood increased with the increased CAP concentration. In the BALF analysis, total cell numbers and the proportion of neutrophils increased with increased CAP concentrations, whilst positive dose-response relationships were also observed for indicators of lung injury. Concentrations of ambient SO_2_, NO_2_, elemental carbon (EC) and organic carbon (OC) were lower, while the concentrations of SO_4_^2−^ were higher during the dust storm compared to values measured during the periods without dust storms.

A contribution from SO_4_^2−^ to pulmonary toxicity was investigated in male mice administered IT with (a) ASD from the Maowusu Desert, China (b) ASD from Shapotou on the fringe of the Tengger Desert, China (c) Shapotou dust plus SO_4_^2−^ (d) AASD collected from the atmosphere of Beijing during a dust storm (Ichinose et al. 2005). Average mass concentration of the tested aerosol in Beijing during the event was 6000 μg/m^3^ (Zhuang et al. 2001). Doses of 50, 100 or 200 μg/mouse were given once a week for four weeks as severe dust storm events occur three or four times a month during spring. Whilst all samples at the higher dose caused neutrophilic inflammation in the bronchi and alveoli, the magnitude was much greater in AASD-treated mice. All particle samples also increased the number of total cells, neutrophils, lymphocytes and eosinophils in BALF and generally exhibited dose dependency. The increased number of neutrophils in BALF correlated with the content of β-glucan in each particle. The numbers of lymphocytes and eosinophils in BALF correlated with the concentration of SO_4_^2−^ in each particle. The pro-inflammatory mediators IL-12, monocyte chemotactic protein (MCP-1), macrophage inflammatory protein (MIP-1α), tumour necrosis factor (TNF-α) and keratinocyte chemoattractant (KC) were greater in the treated mice, including a considerable increase following ADSP at the 200 μg dose. The increased amounts of MIP-1α and TNF-α corresponded to the amount of β-glucan in each particle and that of MCP-1 and IL-12 corresponded to the concentration of SO_4_^2−^.

Yanagisawa et al. used microarray analysis to detect alterations in global gene expression in the murine lung following exposure to ASD sampled from the Tengger Desert (Yanagisawa et al. 2007). The emphasis was placed on the role of microbial materials, by IT administering male ICR mice with either untreated ASD or ASD heated at 360 °C (H-ASD; 250 μg/animal) and thus free of LPS and β-glucan. Asian sand dust, but not H-ASD exposure markedly enhanced inflammatory response-related genes, and this was accompanied by increased expression of pro-inflammatory molecules in lung tissue. Histologic examination showed that neutrophilic lung inflammation was far more prominent in the ASD group than in the H-ASD group.

In that heat removal of microbial components from ASD causes fewer respiratory effects (He et al., 2010, Yanagisawa et al., 2007), prompted an investigation as to whether Asian sand particles can exacerbate pneumonia induced by pathogenic bacteria (He et al. 2012b). Male mice were instilled IT with heat-treated Iki-island AASD (H-AASD) at doses of 50 μg or 200 μg /mouse four times at 2-week intervals in the presence or absence of *Klebsiella pneumonia* (KP) at the last IT instillation. Pathological examinations and cellular profiles of BALF showed that H-AASD exacerbated pneumonia incidence in KP infected mice and increased expression of cytokines (IL-1β, IL-6, IL-12, IFN-γ, TNF-α) and chemokines (KC, MCP-1, MIP-1α) related to KP in BALF. Effects were more enhanced in the higher dose AASD plus KP group. Results of an accompanying *in vitro* study using RAW264.7 cells, prompted speculation that the exacerbation of pneumonia by ASD plus KP was due to the enhanced production of pro-inflammatory mediators via activation of Toll-like receptor 2 (TLR2) and NALP3 (NACHT domain, leucine-rich repeat, and pyrin domain-containing protein 3) inflammasome pathways in alveolar macrophages.

Studies from the collaborating research groups of Hirohisa Takano (National Institute for Environmental studies, Tsukuba) and Misaki Naota (Kyoto University) in Japan, primarily focused on acute and chronic pulmonary toxicity induced in male mice by IT installation of ASD particles (single doses of 50, 200, 800, and 3,000 μg) free from chemical and biological substances. The acute study tested heat sterilized particles from 2 sources: simulated particles collected from the Tennger Desert (CJ-2 particles) and AASD collected from the atmosphere in Tottori, Japan during dust storm events (Naota et al., 2010, Naota et al., 2013). Localized accumulation of the dust particles was observed in the bronchioles and the alveoli and 24 h post exposure, particles caused acute inflammatory changes. The primary inflammatory cells observed around the particles in both BALF and lung tissue were alveolar macrophages and neutrophils and their presence increased in a dose-dependent manner. Cellular degeneration of the alveolar walls and bronchial epithelium was also observed and this was severe at the 2 higher doses. The similar histopathological changes observed with CJ-2 and Tottori samples reflected the similar characteristics of these particles in terms of size and concentrations of elements and minerals (Nishikawa et al. 2000). Proinflammatory cytokines, nitric oxide synthase (iNOS) and copper- and zinc-containing superoxide dismutase (SOD) were observed mainly in the inflammatory cells of the lesions. To study toxicity over time of low (200 and 400 μg) and high doses (800 and 3,000 μg) of CJ-2 particles (again heat treated to remove chemical and biological pollutants), animals were sacrificed at 24 hr, 1 week, or 1, 2, 3 or 4 months after instillation (Naota et al., 2013, Shimada et al., 2015). The acute inflammation observed 24 h was transient, subsiding at 1 week. However at 2, 3 and 4 months, an exacerbation of inflammation, characterised by infiltration of lymphocytes and granulomas with multinucleated giant cells in lung tissues (following both low and high doses) was observed. The size of the granulomatous lesions induced by the high dose gradually increased, with accompanying collagen deposition, over time, possibly a result of altered regulation of the extracellular matrix (Shimada et al. 2015). The same researchers went on to investigate the propensity of ASD induced-acute lung toxicity to enhance translocation of 50 nm gold nanoparticles into the systemic circulation across the damaged air–blood barrier (Rattanapinyopituk et al. 2013). The lungs of male ICR mice instilled IT with 800 μg CJ-2 particles 24 h before instillation of gold nanoparticles exhibited acute lung inflammation consistent with previous findings (Naota et al. 2010). This was accompanied by destruction of the alveolar walls with an increased number of endocytic vesicles containing gold nanoparticles in the cytoplasm of both type I epithelial cells and endothelial cells. In that gold nanoparticles were also found in alveolar macrophages suggests a role for the latter in taking up and eliminating nanoparticles from the alveoli through a phagocytic process. Of note, no gross or histopathological lesions were observed in the systemic organs from mice treated with ASD and gold nanoparticles or mice treated with gold nanoparticles alone.

Zinc has a structural and functional role in a large number of macromolecules. It is essential for over 300 enzymatic reactions necessary for tissue regeneration and repair (Vallee and Falchuk 1993), influences inflammation via the production and signaling of numerous inflammatory cytokine in a variety of cell types (Bao et al., 2008, Bao et al., 2009, Prasad et al., 2007) and is important in airway homeostasis (Kamei et al. 2018). These functions, coupled with zinc deficiency being identified as a significant public health problem (Hambidge and Krebs 2007), prompted a study to assess the effects of low serum zinc on the ASD-induced lung toxicity. Male mice fed diets containing a normal or low content of zinc for 8 weeks were IT instilled with 3000 μg of heat sterilized ASD, followed by sacrifice at 24 h, 2 weeks, and 1, 2 and 3 months after instillation (Shimada et al. 2018). Although the lungs revealed similar patterns of acute and chronic inflammatory changes, they were more prominent and persistent in mice with low serum zinc. Results further suggested a zinc deficiency may induce the modulation of cytokine expression and lysosomal malfunction by phagocytotic and/or autophagic mechanisms.

##### 3.1.2.2 Arizona desert dust

Biological effects of 2 samples of surface sediment collected from separate dust sources in northeastern Arizona desert have been investigated. Mice were IT instilled (100 μg) with Arizona grey sediment, Arizona red sediment, carbon black (CB), SiO_2_ or ambient PM (NIST 1649) (Ghio et al. 2014). Animals showed the greatest lavage concentrations of pro-inflammatory mediators, neutrophils and N-acetyl-beta-D-glucosaminidase and LDH following treatment with SiO_2_ and the desert dusts.

##### 3.1.2.3 Middle East sand particles

Studies have also been designed to better understand the pulmonary response of US and coalition forces deployed to Kuwait, Iraq and Afghanistan where sand dust storms and the movement of troops and equipment can increase airborne PM_10_ to concentrations exceeding 10,000 μg/m^3^ and where annual averages are 100–200 μg/m^3^ (Draxler et al., 2001, Engelbrecht et al., 2009).

Wilfong et al. reported acute and chronic pulmonary responses to Kuwaiti sand from Camp Buerhing (Udairi) relative to SiO_2_ (a known acutely toxic fibrogenic dust) and TiO_2_ (a known low toxicity non-fibrogenic dust) in male rats following a single IT instillation of 1000, 5000 or 10,000 μg (Wilfong et al. 2011). Compared to SiO_2_, results suggest that for acute exposures, Middle East PM_10_ is a nuisance-type dust with relatively low toxicity. To further characterize respiratory toxicity, Dorman et al. looked at whether mainstream cigarette smoke (MSCS) could exacerbate particle-induced effects of Iraqi sand (Dorman et al. 2012). Smoking and other tobacco use among active duty members of the US Military remains higher than that seen in the general population especially during deployment (Poston et al. 2008). Male rats underwent a 6-week nose only inhalation to air or MSCS (3 h/d, 5 d/wk) that included exposure to Iraqi sand collected at Camp Victory near Baghdad or SiO_2_ (1000 μg /m^3^, 19 h/d, 7 d/wk) during the last 2 weeks. Despite elevated concentrations of aluminum, SiO_2_, barium, manganese and vanadium in lung parenchyma in Iraqi sand-exposed rats, indicative of the bioavailability of certain metals, the exposure did not result in alterations in body weight gain, impaired pulmonary function or airway pathology. This minimal toxicological response, limited to mild inflammation in the anterior nose and lung, was also reflected in the results of lung gene expression and proteomics studies. Inhalation of MSCS with or without co-exposure to either Iraqi sand or SiO_2_ resulted in changes consistent with pulmonary inflammation and a stress response and whilst certain histopathologic responses were exacerbated by SiO_2_-MSCS co-exposure, effects were not potentiated in animals exposed to Iraqi sand plus MSCS.

Geographical variation in the respiratory toxicity of Middle Eastern sands has also been studied by evaluating sand particles collected at military bases near Fort Irwin USA, in Iraq (Camp Victory, Taji and Talil), and in Khost, Afghanistan (Taylor et al. 2013). By using aqueous extracts containing a complex mixture of nickel, manganese, vanadium, cadmium, cobalt and chromium, the focus was on role of soluble metals. The relative *in vitro* cytotoxicity of the sand extracts, assessed using replicating rat type II alveolar cell cultures was Afghanistan < Camp Victory & Fort Irwin < Taji & Talil. These results were partially predictive of *in vivo* responses assessed in male rats following IT administration (100 μg per animal). Although the metal content varied between geographic regions, it was not possible to elucidate the individual metal(s) that contributed to the observed toxicity.

##### 3.1.2.4 Summary of effects on lung damage/inflammation

• Positive dose response relationships (inhalation: 316 v 685 μg/m^3^; IT: 50–200 μg /week for 4 weeks) have been observed for both ASD or AASD and inflammatory lung injury in the lower respiratory tract of animal models.
• An increase of white blood cells in peripheral blood may reflect a systemic response following the exposure.
• Studies that have looked at the effects of added SO_4_^2−^, heat sterilized dust particles plus those that analysed microbial content suggest that the differences in the magnitude of inflammatory lung injury depends on the amounts of toxic materials adhered onto the dust particle.
• However, studies have also demonstrated that higher doses of mineralogical components of ASD particles, free from chemical and biological pollutants, cause acute inflammatory changes and degeneration of the structure of the air–blood barrier. Signs of chronic toxicity include collagen deposition associated with granuloma formation.
• Compared to other particles, release of pro-inflammatory mediators and indices of lung damage after exposure of mice to Arizona desert dusts (IT: 100 μg) approached that of silica.
• Ambient ASD (IT: 50 or 200 μg × 4 at weekly or 2 weekly intervals) exacerbates KP-induced pneumonia whilst accompanying *in vitro* studies suggest this is mediated by enhanced production of pro-inflammatory mediators via activation of TLR2 and NALP3 inflammasome pathways in alveolar macrophages.
• Compared to silica, Middle East sands (IT: 10–10,000 μg) have been found to exert a minimal and transient toxicological response in rats, limited to mild pulmonary inflammation. Furthermore, unlike silica, these sand dusts were unable to potentiate the effects of mainstream cigarette smoke.

#### 3.1.3 Allergic respiratory disease

A large tranche of studies into the effects of ASD on allergic respiratory disease originate from the collaborating groups of Takayuki Shibamoto (University of California), Takamichi Ichinose (Oita University of Nursing and Health Sciences, Japan) and Miao He (China Medical University, Shenyang). The focus of this research effort has been on the exacerbating effects of the components of ASD on allergen-induced (a) pathologic changes in respiratory airways, (b) cytological alteration/proinflammatory mediators in BALF and (c) concentrations of IgE and IgG1 antibodies in serum.

##### 3.1.3.1 Biological materials

The contribution of ASD-adhered microbial materials to allergic lung inflammation and possible underlying mechanisms, including those involving toll like receptors, have been documented in several studies (Ichinose et al., 2008a, He et al., 2010, He et al., 2013a, He et al., 2016b, Ren et al., 2014b). Toll like receptor 2 is a receptor for β-glucan or peptidoglycan of Gram-positive bacteria (Beutler 2002) and TLR4 is a receptor for LPS (Schwandner et al. 1999). Myeloid differentiation factor 88 (MyD88), a downstream signaling adapter molecule, is a principal adapter protein and essential for cytokine production in response to TLR ligands (Schnare et al. 2001). Ichinose et al. IT instilled male mice with either heat treated ASD (H-ASD; 100 μg), unheated ASD, ovalbumin (OVA), OVA + H-ASD or OVA + ASD, four times at 2 week intervals (Ichinose et al. 2008a). Asian sand dust (but not H-ASD) increased neutrophils in BALFs along with pro-inflammatory mediators KC, IL-12, IFN-α, RANTES and MIP-α. Both H-ASD and ASD enhanced eosinophil recruitment induced by OVA in the alveoli and in the submucosa of the airway and synergistically increased IL-5, MCP-3 and eotaxin associated with OVA in BALF but the enhancing effects were much greater in ASD than in H-ASD treated animals. The two ASDs also induced the adjuvant effects to specific IgE and IgG1 production by OVA. In the accompanying *in vitro* study using RAW264.7 cells, ASD increased the expression of TLR2 mRNA but not TLR4 mRNA, whilst H-ASD caused no expression of either TLR mRNA. An almost identical set of experiments used AASD collected from Iki-island in Japan, after a massive 3-day dust storm event occurred in East Asia (He et al. 2010). The average density of ambient PM (total suspended particle [TSP]) at this time was 672–796 μg/m^3^ (Sun et al. 2004). In line with previous findings, IT instillation of AASD into male mice enhanced OVA-induced bronchitis and alveolitis, and increased neutrophils along with Th1 relevant cytokines and eosinophil-relevant cytokines and chemokines in whole lung lavage fluid. Again, heat sterilization of the particles to exclude toxic materials caused considerably fewer effects. The accompanying *in vitro* study also reported that AASD increased the expression of TLR2 mRNA, but not TLR4 mRNA, as well as an increased mRNA expression of NALP3, ASC (apoptosis-associated speck-like protein containing a caspase activating and recruitment domain) and IL-1β (He et al. 2010). The aggravating effects of different AASD based on their source regions and passage routes have also been compared (He et al. 2013a). Both dusts were sampled from Fukuoka, Japan. One was transported there from a large and very severe event that originated from the Badanjilin Desert in Inner Mongolia (AASD1), the other from a middle-scale dust event that originated from the Hunshandake desert in northeast China (AASD2). The AASDs contained different amounts of LPS, β-glucan (ASD1 < ASD2) and SiO_2_ (ASD1 > ASD2). Male mice instilled with AASD1 or AASD2 (100 μg/mouse) four times at 2-week intervals exhibited enhanced eosinophil recruitment induced by OVA in the submucosa of the airway, with goblet cell proliferation in the bronchial epithelium. The aggravating effects were more severe in LPS rich AASD2 than in SiO_2_ rich AASD1. Both samples synergistically increased OVA-induced eosinophil-relevant cytokines IL-5, IL-13 (AASD1 < AASD2) and chemokine eotaxin (AASD1 > AASD2) in ΒALF. When WT, TLR 2(−/−), 4(−/−), and MyD88(−/−) BALB/c mice were IT challenged with OVA and/or AASD1, exacerbation of lung eosinophilia, increased Th2 cytokine and eosinophil-relevant chemokine production and induction of serum IgE and IgG were observed (He et al. 2016b). Responses observed in WT mice were similar to those in TLRs 2(−/−) and 4(−/−) but not in MyD88(−/−) mice. Results therefore indicate that ASD exacerbates lung eosinophilia in a MyD88-dependent pathway. Treatment of bone marrow-derived macrophages (BMDMs) from WT, TLR2−/−, TLR4−/− and MyD88−/− BALB/c and WT, TLR2−/−, TLR4−/−, TLR2/4−/− and MyD88−/− C57BL/6J mice with an AASD (20 μg/ml) enhanced the secretion of IL-6, IL-12, TNF-α, MCP-1 and MIP-1α into the culture medium (He et al., 2013a, He et al., 2016b). Cytokine production in BMDMs was higher in ASD-stimulated TLR2(−/−) cells than in TLR4(−/−) cells, whereas it was lower or undetectable in TLR2/4(−/−) and MyD88(−/−) cells, indicating that the MyD88-dependent pathway through TLR4 was the predominant one. Ren et al. have investigated whether the level of LPS contamination in ASD is related to the degree of aggravation of the lung eosinophilia (Ren et al. 2014b). Male BALB/c mice were instilled IT with 12 different testing samples prepared with mixed or individual solutions of naturally occurring LPS (1 ng or 10 ng), H-ASD (100 μg), and OVA. H-ASD enhanced LPS-induced neutrophilic lung inflammation and expression of pro-inflammatory mediators in BALF. In the presence of OVA, LPS increased the level of eosinophils slightly and induced trace levels of Th2 cytokines IL-5 and IL-13 at the levels of 1 ng and 10 ng. In the presence of OVA and H-ASD, LPS induced severe eosinophil infiltration and proliferation of goblet cells in the airways as well as remarkable increases in Th2 cytokines IL-5 and IL-13 in BALF and these responses were more remarkable at 1 ng LPS than at 10 ng. The mixture containing LPS (1 ng) also showed adjuvant activity on OVA-specific IgE and IgG1 production. Accompanying *in vitro* studies again indicated that the aggravation of the allergic lung inflammation by LPS occurs through a TLR4- dependent signaling pathway.

The effects of ASD from 2 sources, with different quantities of β-glucan, on mite allergen (*Dermatophagoides farinae* [*D. farinae*]) induced eosinophilic inflammation in the murine lung have also been investigated (Ichinose et al. 2006). Sand dusts from the Maowusu (SD1; 26.4 pg/mg β-glucan) or Tengger deserts in China (SD2; 12 pg/mg β-glucan) were IT administered alone (100 μg, 4 times at 2 weekly intervals) or in combination with *D. farinae*. Whilst both sand dusts enhanced *D. farinae* -induced eosinophil airway infiltration and goblet-cell proliferation, the degree of eosinophil infiltration induced with SD2 was greater than with SD1, as was the synergistic or cumulative *D. farinae* -induced concentrations of IL-5, eotaxin and MCP-1 in the BALF. However, SD-1 increased the expression of IFN-γ in BALF with or without *D. farinae*, but SD-2 did not. The reduced eosinophil infiltration in the SD-1-treated mice was therefore surmised to be due to suppression of Th-2 cytokines and eotaxin via IFN-γ induced by microbial materials, such as β-glucan.

Τhe exacerbating effect of a combined exposure to zymosan A (ZymA; from the yeast *Saccharomyces cerevisiae*, as a source of β-glucan) and H-ASD (National Institute for Environmental Study No.30 ‘Gobi Kosa Dust’) on OVA-induced murine lung eosinophilia has also been investigated (Sadakane et al. 2016). Male BALB/c mice were repeatedly instilled IT with one of eight immunogenic formulations prepared with mixed or individual solutions of (1) ZymA, (2) H-ASD (100 μg), and (3) OVA. Exposure to ZymA with or without OVA had no effect on most indicators of lung inflammation. Exposure to H-ASD with OVA increased the recruitment of inflammatory cells to the lungs and the serum levels of OVA-specific IgE and IgG1. The combination OVA + ZymA + H-ASD induced a marked recruitment of eosinophils and upregulation of Th2 cytokines (IL-4 and IL-13), IL-6, eotaxin/CCL11 and MCP-3/CCL7 in BALF and OVA-specific IgE in serum. This treatment also induced the most severe pathological changes in the lungs of mice. These researchers then went on to study the effects of co-exposure of LPS and ZymA in exacerbating a response akin to allergic asthma associated with ASD (100 μg) in male BALB/c mice (Sadakane et al. 2019). Exposure to OVA plus LPS enhanced the recruitment of inflammatory cells to the lungs, particularly neutrophils whilst the addition of H-ASD potentiated this effect. Exposure to OVA plus ZymA did not affect most indicators of lung inflammation, whilst adding H-ASD particularly stimulated the recruitment of eosinophils and serum levels of OVA-specific IgE and IgG1 antibodies. Exposure to the full OVA/LPS/ZymA/H-ASD mix affected a few allergic parameters additively or synergistically, however most measured allergic parameters were in line with those observed following exposure to OVA plus LPS plus H-ASD (marked neutrophil recruitment) or OVA plus ZymA plus H-ASD (marked eosinophil recruitment).

Another biogenic agent, *Bjerkandera adusta* (*B. adusta*) is one of the most important etiological fungi associated with chronic cough (Ogawa et al. 2009) and has also been isolated from windborne ASD aerosol (Kobayashi et al. 2010). For these reasons, Liu et al. investigated the exacerbating effects of (a) AASD on *B. adusta*-induced lung inflammation and (b) *B. adusta* plus AASD on OVA-induced murine lung eosinophilia (Liu et al. 2014). *B. adusta* obtained from AASD aerosol was inactivated by formalin and AASD collected from the atmosphere was heated to remove toxic organic substances (H-AASD). Male mice were then instilled IT with 12 different samples prepared with various combinations of *B. adusta*, H-AASD (100 μg) and OVA. H-AASD aggravated the lung eosinophilia and increase in BALF inflammatory cell numbers, pro-inflammatory cytokines and chemokines induced by the fungus alone. A mixture of OVA, H-ASD and *B. adusta* caused the most extreme exacerbation of allergic airway inflammation, consisting of serious fibrous thickening of the subepithelial layer, eosinophil infiltration and proliferation of goblet cells in the airways along with substantial increases of IL-13, eotaxin, IL-5, and MCP-3 in BALF. In similar experiments, He et al. IT instilled male mice with *B. adusta* (0.2 μg or 0.8 μg) in the presence or absence of H-AASD (100 μg), four times at 2-week intervals (M He et al. 2014). Lung eosinophilia caused by *B. adusta* was aggravated by H-ASD with *in vitro* studies suggesting this may be related, at least in part, to the activation of the TLR2–NF-kB signaling pathway in antigen presenting cells.

The role of ASD in aggravating the nasal allergic reaction induced by Japanese cedar pollen (JCP), one of the most common causes of pollinosis in Japan has also been investigated (Ichinose et al. 2009). Male guinea pigs were administered ASD (300 or 600 μg), JCP or JCP + ASD into their nasal cavities at seven weekly intervals. Whilst ASD alone did not exhibit any effects, an adjuvant effect on allergic rhinitis induced by JCP was evident. Asian sand dust enhanced the JCP-associated nasal obstructing response, but not the number of sneezes or amount of nasal secretions. Analysis of nasal cavity lavage fluids showed that ASD enhanced JCP-associated cysteinyl leukotriene and histamine production and eosinophil number. Asian sand dust also enhanced JCP-associated eosinophil recruitment in the nasal mucosa, goblet cell proliferation in the nasal epithelium and total IgE in serum.

##### 3.1.3.2 Sulphate

Hiyoshi et al. investigated the effects of either ASD alone or ASD plus SO_4_^2−^ toward allergic respiratory disease (Hiyoshi et al. 2005). Particles were collected from surface soils in Shapotou and IT administered to male mice at a dose of 100 μg per animal. Asian sand dust enhanced OVA-induced eosinophil recruitment in the alveoli and submucosa of the airway plus goblet cell proliferation in the bronchial epithelium. A further effect on the studied endpoints by the addition of SO_4_^2−^ was not observed, suggesting that the inflammatory response caused was due to the mineral particles and/or microbiological materials.

##### 3.1.3.3 Mineral elements

Studies have also focused their attention on the mineral elements of dust particles by IT administering male mice with ASD collected from the surface soils of Shapotou, Arizona sand dust, amorphous SiO_2_ (99%) or aluminum oxide (Al_2_O_3_: 99%), with or without OVA (Ichinose et al. 2008b). The content of minerals ranged from 0.7% (TiO_2_) to 60% (SiO_2_) in ASD and from 0.5% (TiO_2_) to 76% (SiO_2_) in Arizona sand dust. The toxic materials adsorbed onto ASD and Arizona sand dust were inactivated by heat-treatment and the dose for all mineral samples was 100 μg per animal 4 times at 2 weekly intervals. The order of potency in enhancing eosinophil number and chemical mediators in BALF was OVA + Al_2_O_3_ < OVA + ASD < OVA + Arizona sand dust < OVA + SiO_2_.

##### 3.1.3.4 Organic chemicals

It is possible that desert dust-bound organic chemicals, formed from the combustion of fossil fuels in industrialised regions, contribute to the aggravation of allergic lung inflammation. To this end, Ren et al., 2014a, Ren et al., 2014b investigated the exacerbating effects of the Tar fraction of ASD collected from the atmosphere in Fukuoka on OVA-induced lung eosinophilia (Ren et al. 2014a). Several PAHs at high concentrations were detected in the Tar fraction including fluoranthene (217 μg/g), benzo[e]pyrene (174 μg/g) and indeno[1,2,3-cd]pyrene (122 μg/g). The concentration of benzo[a]pyrene (B(a)P), the most potent carcinogen, was 34.6 μg/g. Male mice were instilled IT with 12 different test samples prepared with Tar (1 μg and 5 μg), H-ASD (100 μg/animal; collected from the Gobi desert) and OVA. Whilst pathological changes caused by H-ASD + OVA were relatively small, the addition of low concentrations (1 μg) of Tar induced severe eosinophil infiltration and proliferation of goblet cells in the airways and significantly increased Th2 cytokines in BALF. The mixture also showed an adjuvant effect on OVA-specific IgG1 production.

##### 3.1.3.5 Urban particulate matter

Analysis of urban PM_2.5_ samples (U-PM_2.5_) collected during hazy weather in a Shenyang, China and fine particles (AASD-PM_2.5_) collected during a dust storm event in Fukuoka revealed that the amounts of β-glucan and mineral components were higher in AASD-PM_2.5_ and that organic chemicals including PAHs were higher in U-PM2.5 (He et al. 2016a). Observations of male mice IT instilled with either particle, with or without OVA, indicated that an exacerbation of lung eosinophilia by both types of PM_2.5_ may be due to activation of a Th2-associated immune response and induced M2 macrophages. Furthermore, the allergic inflammatory responses were greater in microbial element (β-glucan)-rich ASD-PM_2.5_ than in organic chemical-rich U-PM_2.5_. The role of oxidative stress, and particularly a contribution from the Fe content of desert dust particles, in the exacerbation of allergen‐induced lung eosinophilia has also been investigated (He et al. 2019). Whilst the concentration of Fe in urban PM_2.5_ and ASD were almost the same, concentrations of Pb, Cu, As, Ni, Cr, Mo, Sb, Co, Se and Cd were greater in PM_2.5_. Male BALB/c mice were IT instilled with OVA alone or a mixed solution of LPS and ASD (no. 30 “Gobi Kosa Dust”) or urban PM_2.5_ with/without chelator deferoxamine (DFO) or oxidative stress scavenger N-acetylcysteine (NAC). The challenge with OVA plus LPS and either urban PM_2.5_ or ASD exacerbated OVA-induced lung eosinophilia along with T-helper 2 cytokine and eosinophil-relevant chemokine production in BALF as well as the production of OVA-specific IgE in serum. Whereas LPS plus PM_2.5_ with NAC tended to reduce the lung eosinophilia, LPS + PM_2.5_ with DFO had no effect. NAC moderately reduced the lung eosinophilia following LPS plus ASD but this was drastically reduced with DFO suggesting that Fe and oxidative stress are at least partly involved in the enhanced lung eosinophilia caused by LPS with ASD.

##### 3.1.3.6 Timing of exposure to sand dust and antigen

The influence of exposure timing on the aggravating effect of ASD on allergen-induced eosinophilic inflammation has also been investigated (He et al. 2012a). Male mice were instilled IT with OVA four times at 2-week intervals, performing simultaneous IT administration of OVA and AASD (OVA + ASD sim) at the last OVA treatment or IT administration with AASD 1 day before (OVA + AASD pre) or after (OVA + AASD post) the last OVA treatment. Whilst all three treatments aggravated allergic lung inflammation, the order of the potency of overall aggravation was OVA + AASD pre < OVA + AASD post < OVA + AASD sim. These workers went on to employ two time-course studies (6 weeks and 14 weeks) to investigate a series of manifestations in lung eosinophilia caused by IT co-exposure to Iki-island AASD and OVA (He et al. 2013b). The design was chosen to mimic Asian dust events that intermittently occur from mid-February to May. Male mice were instilled IT with 100 μg of ASD per mouse four times (over 6 weeks) or eight times (over 14 weeks) at 2-week intervals (total dose of 400 μg or 800 μg/mouse) with or without OVA. Four-time co-exposure to OVA and AASD was found to aggravate allergic airway inflammation. An increased expression of Th2-cytokine IL-13 and eosinophil-relevant cytokine/chemokines IL-5, eotaxin and MCP-3 in BALF, was accompanied by fibrous thickening of the subepithelial layer. The eight-time co-exposure attenuated these changes along with a significant increase of transforming growth factor (TGF-β1) in BALF. The adjuvant effects of AASD toward IgG1 and IgE production in sera were however, still evident in the eight-time co-exposures.

##### 3.1.3.7 Mucin production

The Korean Meteorological Association issues Asian dust warnings when the hourly averaged dust (PM_10_) concentration is expected to exceed 400 μm/m^2^ for longer than 2 h. Studies using AASD collected from Incheon City during such warnings have focused on mucin production (Jung et al., 2012, Kang et al., 2012). Mucins are the highly glycosylated proteins responsible for the viscoelastic properties of mucus (Williams et al. 2006). The production of mucus in the respiratory tract provides a barrier between the external environment and the cellular components of the epithelial layer. The appropriate quantity and qualitative characteristics of mucus are hence an important host defense mechanism against airborne pathogens as well as providing protection against chemical and mechanical damage. On the other hand, mucus hypersecretion is one of the major symptoms and signs of upper and lower airway inflammation and causes substantial morbidity and mortality in airway diseases (Ali and Pearson 2007). Male BALB/c mice sensitized with OVA were treated with AASD at 10,000 μg/ml via a nebulizer for 15 min each day for 7 days – a dose calculated to be very similar to the total amount inhaled by humans in an atmosphere during ASD warnings (Jung et al., 2012, Kang et al., 2012). Animals exhibited higher numbers of eosinophils and Periodic Acid Schiff (PAS)-positive cells in the nasal epithelial tissues 1–2 weeks post exposure and at 2 weeks, greater numbers of MUC5AC- and TGF-α-immunopositive cells were observed. The airborne Asian sand dust also increased OVA-specific serum IgE levels and IL-4 and IL-5 concentrations in BALF and cytokine-positive cells lung tissue. An *in vitro* component to the study by Jung et al. also reported significantly higher numbers of MUC5AC- and PAS-positive cells and increased MUC5AC mRNA expression among human NCI-H292 pulmonary mucoepidermoid carcinoma cells treated with AASD (10, 100, 250, or 500 μg/ml) (Jung et al. 2012).

##### 3.1.3.8 Summary of effects on allergic respiratory disease

• Virgin and wind-borne ASD (IT: 100–200 μg single dose or × 4 at 2 week intervals) aggravates antigen-related lung eosinophilia via increases in Th2-mediated cytokines and antigen-specific immunoglobulin in murine models of asthma after IT instillation.
• The aggravated lung eosinophilia by ASD may be due to an induction of TLR signaling via a MyD88-dependent signaling pathway.
• The simultaneous exposure of ASD and OVA aggravates lung eosinophilia remarkably compared with administration of ASD either one day before or after OVA treatment.
• Whilst four-time sensitization of OVA with ASD aggravates allergic inflammation along with fibrous thickening of the subepithelial layer in the airway, eight-time sensitization attenuates these changes. Results suggest that the suppressive responses are caused by TGF-β1, which may have an important role in the self-defense reaction for repairing the severe airway injury and weakening the eosinophilic inflammation enhanced by ASD at an early stage.
• The aggravating effects of desert dusts have been found to be dependent on the SiO_2_ content, suggesting that enhancement may be mediated, at least partly, due to the mineral elements.
• Studies (a) employing heat treatment to exclude toxic materials adsorbed onto ASD, (b) analysing microbial content of dust partcles plus (c) those adopting co-exposures (ie ASD plus microbes) suggest that microbial materials such as β-glucan and LPS adsorbed onto ASD may contribute to the exacerbation of lung eosinophilia.
• Various microbial elements may play different roles in allergic airway inflammation with ASD, in that LPS‐rich ASD induces neutrophilic inflammation in the lower respiratory tract and lungs, while β‐glucan‐rich ASD induces eosinophilic inflammation.
• Potentiating effects of ASD have been observed in lung inflammation caused by Gram negative bacteria, *B. adusta* and *D. farinae*.
• Whilst results suggest that chemical species, such as sulphate, are not involved in the aggravating effects of lung eosinophilia and allergic diseases, aggravation by Tar might be caused by PAHs.
• A study comparing exposure to organic chemical-rich urban-PM_2.5_ versus microbial element-rich desert- PM_2.5_ suggests that the latter causes a greater allergic inflammatory response.
• The presence of Fe in dust particles causing oxidative stress are at least partly involved in lung eosinophilia exacerbation caused by LPS and ASD.
• Ambient ASD can aggravate airway disease by activating inflammatory reactions through increased mucus secretion.

#### 3.1.4 Systemic toxicology

One of the few animal studies to employ an inhalation exposure examined systemic toxicity (respiratory, cardiovascular, endocrine and digestive systems) of PM collected from the Alxa Plateau of Inner Mongolia by simulating a real dust storm environment using a wind tunnel system (Cao et al. 2018). Rats were exposed to 9000 μg/m^3^ for 5 h each day and sacrificed on day 45, 90, 135 or 180. Repeated exposure at these high concentrations of dust storm PM was associated with an increase in circulating inflammatory cytokines and enzymes, a decrease in antioxidant blood profile (SOD and glutathione [GSH]; increased iNOS) and pathological changes in the lung, kidney and spleen but not in the heart, liver, stomach, lung and thymus.

#### 3.1.5 Lymphoid organs

Despite the wealth of animal studies investigating the way in which ASD induces pulmonary inflammation, a limited understanding exists regarding the inflammatory effects of ASD on other organs. Since immune cells residing in peripheral lymphoid organs and circulating to sites of inflammation play important roles in allergy, the effects of AASD on splenic events have been investigated (Song et al., 2015, Song et al., 2019). Song et al (2015) showed that administration of AASD (IT 100 μg; heat treated and untreated), collected from the atmosphere at Kitakyushu in Japan, to ICR mice induced pulmonary inflammation (increased TNF-α in BALF) on day 1 but not day 3, and modified peripheral lymphoid splenocytes on day 3 but not day 1, suggesting a triggering of systemic inflammation. Effects on splenocytes were an increased mitogen-induced IL-2, TNF-α and IL-6 production as well as enhanced activation of NF-κB in CD4^+^ and CD11b^+^ cells. These researchers went on to study effects of a subchronic exposure to AASD by administering mice with AASD once every 2 weeks for 8 weeks (Song et al. 2019). The results of an elegant series of experiments using wild-type and knockout (TLR2^−/−^, TLR4^−/−^ and MyD88^−/−^) mice indicate that the AASD particle (as opposed to particle constituents) induced splenic inflammation via TLR4-MyD88 signaling.

### 3.2 *IN VITRO* STUDIES (see Table S2)

#### 3.2.1 Dosage

Whilst extrapolation from *in vitro* concentrations to human exposures is challenging, attempts have been made in the studies described in this section to compare chosen doses with those that reflect a typical blowing sand day. An example is the study by Geng et al. (2005), summarized below, that reported normal weather and blowing sand weather PM_2.5_ concentrations of approximately 60 and 190 μg/m^3^. An “equivalent” human 24 h dose (EHD) was estimated for the hypothetical case of a person exposed to a particle concentration of 60 μg/m^3^ at a ventilation rate of 15 l/min and an estimated 15% particle deposition fraction for the lung parenchyma (Kleinman et al. 2003) as follows: EHD = 60 μg/m^3^ × 15 l/min × 24 h × 0.15 = 194.4 μg. However since the mass concentration of blowing sand PM_2.5_ was approximately three times that of normal PM_2.5_, the PM_2.5_ dosages treating alveolar macrophages (2.4 × 10^6^ cells) were classified as 33 μg/ml (low dose), 100 μg/ml (mid dose) and 300 μg/ml (high dose). These calculations go some way in helping to put into context the doses employed in this section.

#### 3.2.2 Respiratory and immune systems

##### 3.2.2.1 Epithelial cell activation and eosinophil migration

To determine the effect of ASD on lower airway epithelial inflammation and eosinophil recruitment, Shin et al. investigated effects on the activation of bronchial epithelial cells (BEAS-2B) and migration of eosinophils (Shin et al. 2013). Three forms of ASD/AASD were tested (10–100 μg/ml): dust collected from the surface soil of the Gobi Desert (GBD; <50 μm in particle diameter), atmospheric PM collected during an ASD event period in Incheon, Korea (PM_50_) and a smaller size, heat-treated equivalent of the latter (PM_10_). Among the three samples, PM_10_ and PM_50_ enhanced the production of IL-6, IL-8 and RANTES whilst GBD enhanced production of only IL-6. Furthermore, when BEAS-2B cells were stimulated with the 2 atmospheric PMs, the conditioned media enhanced eosinophils migration by more than double. In contrast, GBD did not influence eosinophil migration, suggesting that AASD containing smaller particles and air pollutants might be key in exacerbating the inflammatory process of bronchial tissue.

Honda et al. 2014 examined the effects of 2 types of AASD particles (AASD1 [plus H-AASD1] and AASD2 transported from Inner Mongolia and northeast China respectively) containing different amounts of chemical elements and microbes (Honda et al. 2014). Effects (at 3, 30 or 90 μg/ml) on the respiratory and immune system were evaluated using human airway epithelial cells, BMD antigen presenting cells (APCs) and splenocytes from atopic prone NC/Nga mice. All AASD samples dose dependently reduced viability of airway epithelial cells suggesting that a physical stimulation is caused by ASD itself, components of ASD themselves or heat-resistant substances adhered to ASD. Non-heated AASD exhibited a dose-dependent increase in the expression of IL-6, IL-8 and ICAM-1 (AASD1 > AASD2). In contrast, H-AASD did not change most of these biomarkers. Non-heated AASD also increased protein expression of DEC205 on APCs and the proliferation of splenocytes, whereas H-AASD did not. Despite evidence of greater activating effects of AASD1, these particles contained lower concentrations of LPS and β-glucan. Το further investigate the responsible ASD factors and underlying mechanisms that lead to respiratory and immune responses, the same workers focused their attention on contributions from *B. adusta* and B(a)P (Honda et al. 2017). In the same collection of cells, both *B. adusta* and B(a)P in the presence and absence of H-ASD increased the expression of cell surface molecules on APCs and the expressions induced by *B. adusta* were higher than those induced by B(a)P. There were no remarkable effects on the activation of splenocytes or the proinflammatory responses in airway epithelial cells.

##### 3.2.2.2 Mast cell granulation and cytokine release

Since mast cells and basophils play keys role in the pathogenesis of allergic disorders, rat basophilic leukemia cells have been used to study effects of ASD on chemical mediator release and cytokine production. Dust samples containing different concentrations of chemical and biological constituents were collected from three sites (Naha, Fukuoka and Tsukuba) in Japan during an Asian dust storm event (Yamada et al. 2012). Exposure enhanced β-hexosaminidase release and TNF-α production and from differences in the concentrations of chemical and biological constituents between samples, it was concluded that these effects may be dependent on endotoxin, *Cryptomeria japonica* pollen (Cry j 1) and other allergens present in the dust extract.

##### 3.2.2.3 Rhinovirusinfection

One of the most common respiratory illness is the common cold caused by rhinovirus (RV) infection (Gwaltney et al. 1966). The latter, in turn, has a prominent role in both upper and lower respiratory tract disease (Greenberg, 2003, Proud and Chow, 2006). Yeo et al. investigated whether AASD (collected from outside of Gachon University building during ASD warnings) may potentiate common cold symptoms associated with RV infection in human nasal epithelial cells (Yeo et al. 2010). Cells were treated with AASD (10–500 μg/ml) for 3 days with or without RV. Dust particles were found to significantly increase RV replication as well as concentrations of RV-induced IFN-γ, IL-1β, IL-6, and IL-8 (the main inflammatory mediators in the pathogenesis of colds caused by RV infection) (van Kempen et al. 1999) mRNA and protein secretion.

##### 3.2.2.4 Mucin/mucus and salvia

The effects of ASD (0–250 μg/ml; 72 h) on the inflammatory process and mucin gene expression have been investigated in nasal polyp epithelial cells (Kim et al. 2011). Cytokine production (IL-8 and GM-CSF but not IL-6) was highest at 100 μg/ml, whilst MUC4 and MUC5B mRNA expression was significantly increased at 10 and 50 μg/ml of ASD. Cytotoxic effects were insignificant. Choi et al. have also focused on signaling pathways of ASD induced mucin expression, in upper and lower airway epithelial cells (Choi et al. 2015). Dust samples (40 μg/ml) increased expression of MUC8, MUC5B and TLR4 (but not TLR2) and activated phosphorylation of extracellular signal-regulated kinase 1/2 (ERK1/2) and p38 mitogen-activated protein kinase (MAPK). U0126 (ERK1/2 MAPK inhibitor) and SB203580 (p38 MAPK inhibitor) attenuated ASD-induced MUC8 and MUC5B expressions, as did knockdowns (by siRNA) of ERK2 and p38 MAPK. Phosphorylations of ERK1/2 and p38 MAPK were also blocked by knockdown of TLR4.

The main role of saliva is to initiate the enzymatic degradation of nutrients however it also protects and lubricates the soft and hard tissues in the oral cavity against mechanical, chemical and thermal irritation. The protection and lubrication functions of mucus and saliva are closely linked to their rheological properties, which in turn are determined by chemical composition, physical parameters, health, age, sex or activity. Artifical mucus and saliva models exposed to Arizona transported desert dust particles (0.06 g/l and 6 g/l) increases viscosity in a dose dependent manner (Penconek et al. 2019). However the presence of particles at a concentration of 6 g/l in mucus had no significant effects on the diffusion of the fluorescent marker through the mucus layer (an indicator of changes in mucus protective properties) implying that the protective function of mucus had not been disturbed.

##### 3.2.2.5 Summary of effects on respiratory and immune endpoints

• Ambient ASD containing smaller particles and air pollutants stimulates airway epithelial cells, enhancing the production of inflammatory mediators and tissue eosinophilia.
• Ambient ASD induces the maturation and activation of bone marrow-derived APCs and increases the proliferation of splenocytes.
• Results suggest that *B. adusta* rather than BaP related to ASD contributes to the activation of the immune system via APCs.
• Data suggests that desert sand dust rapidly enhances chemical mediator release in basophilic cells. This process may depend on adhered allergen content such as endotoxin and Cry j 1.
• Ambient ASD may potentiate common cold symptoms associated with RV infection not only by enhancing the inflammatory response (increased IFNγ, IL-1β, IL-6, IL-8 secretion in primary nasal epithelial cells to a greater extent than either agent alone), but also by increasing viral replication.
• Asian sand dust induces MUC8 and MUC5B expressions via TLR4-dependent ERK2 and p38 MAPK signaling pathway in human upper and lower airway epithelial cells.
• Although the presence of ambient sand dust in saliva and mucus models increased their apparent viscosity, no significant effects were observed on the diffusion of a fluorescent marker through mucus layer, implying that the protective function of mucus would not be disturbed.

#### 3.2.3 Cytotoxicity & oxidative/nitrosative stress

Early research into the contribution that oxidative stress may play in desert dust-cytotoxicity quantified 8-oxo-dG as a measure of direct ROS generation, in response to particulate exposures (100–1000 μg/ml) to either free 2′-deoxyguanosine (dG) or calf thymus DNA (Prahalad et al. 2001). In addition to Arizona desert dust, coal fly ash (CFA), oil fly ash (OFA and ROFA) and ambient air particulates (SRM and DUSS) were tested. These were selected on the basis of concentrations of water-soluble metals (V, Ni, Fe) in OFA and ROFA compared to insoluble constituents (Si, Al, Fe) in desert dust, urban particulates and CFA. Overall, damage to the cell-free systems was consistent with the concentration of water-soluble rather the total metal content of the particle. For example, using calf thymus DNA all the particles induced 8-oxo-dG in a pattern similar to that observed for dG hydroxylation, with OFA, ROFA, SRM and DUSS producing significant increases. The systems exposed to Arizona desert dust and CFA showed slightly elevated but not significant effects.

Release of reactive oxygen and nitrogen species in association with cytotoxicity in alveolar epithelial cell lines exposed to yellow sand (China Loess, obtained from the Gunsu Province of China), SiO_2_ or TiO_2_ (100 μg/cm^2^) has been investigated. (Kim et al. 2003). Cell viability in yellow sand-stimulated cells was higher than that in SiO_2_-stimulated cells and lower than that in TiO_2_-stimulated cells. Effects of the particles on intracellular calcium concentrations were very similar to those for cell viability. All particles induced the generation of hydrogen peroxide with no clear difference in potency. In contrast, yellow sand showed high Fenton activity, SiO_2_ slight activity whilst TiO_2_ did not change activity. All particles induced nitrite formation (SiO_2_ > TiO_2_ > yellow sand) whilst SiO_2_ and yellow sand also increased the release of TNF-α (SiO_2_ > yellow sand). Others have investigated the influences of blowing sand PM_2.5_ on rat alveolar macrophage plasma membrane permeability/fluidity and intracellular calcium ion concentration (Geng et al., 2005, Geng et al., 2006). Cells were treated with normal PM_2.5_ (collected on sunny, non-blowing sand days) and blowing sand PM_2.5_ collected in Chinese cities (Wuwei, Gansu Province and Baotou, Inner Mongolia). Doses were chosen to reflect a typical blowing sand day (33, 100 & 300 μg/ml; 4 h). All particles induced oxidative stress (decline in cellular GSH and increased malondialdehyde concentrations), increased plasma membrane fragility and elevated intracellular calcium levels in a dose-dependent manner – effects that ultimately led to cytotoxicity and cell death. The toxic effects of normal and blowing sand PM_2.5_ (and also the water soluble fractions in the case of the Bautou city study) were relative to treatment dosages but not to dust types, suggesting the blowing sand PM_2.5_ whose airborne mass concentrations were much higher should be more harmful.

The role of oxidative stress and subsequent cell signaling in the inflammatory effects of desert dust was investigated by Ghio and colleagues (Ghio et al. 2014). Two samples of surface sediment collected from separate dust sources in northeastern Arizona were compared with CB, SiO_2_ and NIST 1649. Characterization of the two desert dusts confirmed that their particles were essentially inorganic with little metal and therefore similar to most North American and global dusts. Exposed respiratory epithelial cells showed significant cytotoxicity and apoptosis. In addition, oxidant generation, activation of MAP kinases and release of pro-inflammatory mediators (TNF-α, IFN-γ, IL-1β, IL-6) were demonstrated. Cell oxidant generation and changes in RNA for SOD-1, heme oxygenase and cyclooxygenase were greatest following exposures to SiO_2_ and the desert dusts. The greatest capacity for MAP kinase activation was shown by the desert dust.

Another approach that has been undertaken to investigate a role for oxidative stress in mediating the toxic effects of ASD is to measure the capacity of the sand particles to cause damaging oxidative reactions (ie the oxidative potential [OP] of the particle). Using the a-cellular dithiothreitol (DTT) assay, the OP of the water-soluble fraction of PM_2.5_ and PM_10_ were evaluated at an urban background site in Southern Italy (Chirizzi et al. 2017). Results were compared during Saharan dust outbreak events with standard samples characterised by concentrations similar to the yearly averages as well as with high carbon samples associated to combustion sources (mainly road traffic and biomass burning). DTT activity normalized by sampled air volume (DTT_V_), an indicator of personal exposure to reactive oxygen species, at a specific site was reported. DTT_v_ activity was larger for PM_2.5_ compared to the coarse fraction (PM_2.5-10_). Moreover, DTT_v_ activity of the high carbon group was more than two times greater than that during Saharan dust outbreak events (especially for PM_10_), which in turn was comparable with that of the standard samples. The OP of airborne PM in Beirut that is influenced by dust events originating in the Sahara and Arabian deserts has also been examined (Lovett et al. 2018). Segregated fine (<2.5 μm) and coarse (2.5–10 μm) PM samples collected during dust events, as well as during non-dust periods, were analyzed for chemical composition and OP using the alveolar macrophage assay. Oxidative potential of Beirut's urban PM during non-dust periods was much higher than during dust episodes for fine PM. The OP of coarse PM was slightly higher during dust days. Findings also indicated that tracers of tailpipe emissions (i.e., EC and OC), non-tailpipe emissions (i.e., heavy metals including Cu, Zn, As, Cd, and Pb), and secondary organic aerosols (i.e., water-soluble organic carbon) were significantly associated with the OP of PM during dust days and non-dust periods. However, the contribution of desert dust aerosols to Beirut's indigenous PM composition did not exacerbate its OP as indicated by the negative correlations between the OP of PM and the concentrations of crustal elements that were enriched during the dust days.

To test the hypothesis that the ability of dust sand to act as a “carrier” during transportation is a determinant of ultimate toxicity, effects of local pollution emissions in 2 downwind cities (Xi’an and Beijing) of the Tengger desert on PM_2.5_ bioreactivity during dust periods have been evaluated (Ho et al. 2019). Particulate samples were collected from the cities during a non-dust day, a pollution episode and 2 dust storm periods (DS1 and DS2). Workers observed a significant decrease in cell viability and an increase in LDH in human alveolar epithelial cells after exposure to PM_2.5_ (50 μg/ml) during a pollution episode and DS-1 in Xi'an and Beijing compared to Tengger Desert PM_2.5_. Using positive matrix factorization to identify pollution emission sources, cell viability and LDH were correlated with PM_2.5_ from biomass and industry during dust storms in Xi'an, whereas vehicle emissions contributed to LDH during dust storms in Beijing. During DS-1, OC, EC, Cl^−^, K^+^, Mg^2+^, Ca, Ti, Mn, Fe, Zn, and Pb were correlated with cell viability and LDH for industrial emissions in Xi'an, whilst OC, EC, SO_4_^2−,^ S, Ti, Mn, and Fe were correlated with LDH for vehicle emissions in Beijing. Notably, desert dust per se was not significantly associated with cell viability or LDH in Xi'an or Beijing during the study periods. This may have been the consequence of higher contributions of local pollutants than the dust itself.

##### 3.2.3.1 Summary of cytotoxicity & oxidative/nitrosative stress

• Some observations suggest that desert sand dust can induce cytotoxicity, that ROS, Fenton activity and RNS might be involved and that its potency appears to be lower than SiO_2_ and OFA. Other studies have shown that the capacity of desert dusts to influence oxidative stress and release of pro-inflammatory mediators is comparable to SiO_2_.
• Although studies indicate no differences between the effects of normal urban PM_2.5_ and blowing sand PM_2.5_ at the same treatment dosages, blowing sand PM_2.5_ should be more harmful in real world conditions since the airborne PM_2.5_ mass concentration is much higher when blowing sand occurs.
• Aerosols generated during dust events have a lower OP compared to combustion-generated PM sampled during non-dust periods.
• The significant amounts of suspended desert sand dust during storm periods may provide a platform to intermix with chemicals on its surfaces, thereby increasing the bioreactivity of PM_2.5_ during dust storm episodes.

#### 3.2.4 Mineral dust surface reactions

Atmospheric nitrated polycyclic aromatic hydrocarbons (NPAHs) have been shown to have adverse health effects such as carcinogenicity (Durant et al., 1996, Patton et al., 1986). They are produced in part, through nitration reactions of parent PAHs in the atmosphere. Some types of NPAHs are formed via gas-phase reactions of semi-volatile PAHs and then subsequently deposit on airborne particulates. One of the most abundant NPAHs is 1-nitropyrene (1-NP), formed by the reaction of pyrene (Py) with gaseous NO_2_ on substrates including graphite and metal oxides (Esteve et al., 2004, Wang et al., 2000). Working on the hypothesis that formation of NPAHs on natural mineral dust could be particularly important owing to surface complexity and reactivity, the effects of (i) authentic mineral dust on the formation of 1-NP from Py and NO_2_ and (ii) heavy dust storms on ambient particle-associated 1-NP in Beijing, China have been examined (Kameda et al. 2016). Results of both studies indicated that mineral dust aerosols dramatically increase the conversion of Py to toxic 1-NP. Whilst the kinetic experiments demonstrated that the reaction is accelerated on acidic surfaces of mineral dust, particularly on those of clay minerals, concentrations of ambient particle-associated NPAHs in Beijing were found to significantly increase during heavy dust storms. In summary, these results suggest that mineral dust surface reactions are an unrecognized source of toxic organic chemicals in the atmosphere and have the potential to enhance the toxicity of mineral dust aerosols in urban environments.

## 4 Discussion

The toxicological literature contains a large number of animal and *in vitro* studies that have investigated intermediate endpoints and mechanisms underlying health effects of desert sand dust and in main, have used doses that reflect or at least approach real world exposures during a dust event. Experimental studies relevant to epidemiological evidence of an association between desert dust and respiratory morbidity have demonstrated that single and repeated airway exposure of mice to ASD or AASD induces inflammatory lung injury in the lower respiratory tract (Lei et al., 2004, Ichinose et al., 2005, Naota et al., 2010) as well as exacerbating KP-induced pneumonia (He et al 2012b). The aggravating effects on allergen (and particularly OVA)-induced lung eosinophilia have been extensively investigated in murine models of asthma and have demonstrated that this is orchestrated by cytokines, chemokines and antigen-specific immunoglobulin potentially via a TLR/MyD88 signaling pathway (Ichinose et al., 2008a, He et al., 2010, He et al., 2013a, He et al., 2016b). Asian sand dust has also been demonstrated to aggravate JCP-induced allergic rhinitis in guinea pigs (Ichinose et al. 2009). *In vitro* studies confirm potential involvement of ASD in exacerbating the inflammatory process of bronchial tissue and asthmatic symptoms through the production of inflammatory mediators and tissue eosinophilia via TLR/MyD88 signaling pathways (Ichinose et al., 2008a, He et al., 2013a, He et al., 2016a). Results are also consistent with the idea that RV-infected patients could be expected to have more severe viral common cold symptoms during periods coinciding with ASD events (Yeo et al., 2010). These findings go some way in clarifying the effects of atmospheric desert dust on the upper and lower human respiratory system.

Despite the large literature base (a) describing the multifaceted nature of effects of ambient PM, and particularly traffic-related particles, on the cardiovascular system (Miller and Newby 2019) and (b) supporting a role for enhanced oxidative stress as a crucial underlying mechanism (Kelly and Fussell 2017), this research effort has not yet been extended to desert sand dust. It is noteworthy however that research on urban PM initially concentrated on respiratory endpoints prior to subsequent foci on mechanisms underlying detrimental effects of particulate air pollution on cardiometabolic health as well as birth outcomes and cognitive function (HEI 2010). Whilst *in vitro* studies suggest that oxidative imbalances may be involved in dust induced cytotoxicity and inflammation, results are conflicting as to whether the capacity of desert dusts to induce oxidative stress is comparable or lower than SiO_2_ (Prahalad et al., 2001, Ghio et al., 2014). Furthermore, aerosols generated during dust events do seem to have a lower OP compared to combustion-generated PM sampled during non-dust periods (Chirizzi et al., 2017, Lovett et al., 2018).

Asian sand dust particles collected from surface soils are composed mainly of silicon, aluminum, calcium and iron but during long-range transportation they become laced with industrial pollutants formed from fossil fuel combustion such as PAHs (Tamamura et al. 2007) nitrates and SO_4_^2−^ (Yu et al. 2020). Microorganisms, such fungal spores and their walls and the lipopolysaccharides of gram-negative bacteria, are more likely to travel with rather than on dust during transportation. The toxicity of these various components of the ASD aerosol have therefore been considered in the context of their effect on human health. Exposure studies that have heated dust particles at 360 °C to eliminate organic substances and chemicals (Ichinose et al., 2005, Ichinose et al., 2008a), sampled AASD of differing compositions (Ichinose et al., 2006, He et al., 2013a) or looked at the effects of added materials (Hiyoshi et al., 2005, Ichinose et al., 2005, Liu et al., 2014, Ren et al., 2014a, Sadakane et al., 2019) suggest that materials adsorbed onto dust particles are probably implicated in the pathogenesis of human respiratory disorders during a dust event. Whilst the responsible factors have not been definitively defined, evidence points to PM-bound trace microbial elements and PAHs rather than SO_4_^2−^, possibly since most dust particles contain calcium with which SO_4_^2−^ form gypsum. Evidence also exists for differential toxicity of adhered components in that Ichinose et al. (2005) reported that increased neutrophils, MIP-1α and TNF-α in BALF corresponded to the content of β-glucan in each particle whilst lymphocytes, eosinophils MCP-1 and IL-12 in BALF correlated with the concentration of SO_4_^2−^ in each particle. Furthermore, studies have demonstrated that the desert sand dust particle itself, rather than its constituents, can cause acute inflammatory changes and degeneration of the structure of the air–blood barrier (Naota et al., 2010, Naota et al., 2013) plus that the aggravating effects are dependent on SiO_2_ content (Ichinose et al 2008b). Together, these findings suggest that in addition to the involvement of adhered chemical and biological pollutants, mineralogical components are also candidate activators of immune and toxicological responses.

The relative toxicity of desert sand dust, compared to both the acutely toxic SiO_2_ and urban PM has also been investigated. Of interest, whilst the release of pro-inflammatory mediators and indices of lung damage after exposure of mice to Arizona desert dusts approached that of SiO_2_ (Ichinose et al., 2008b, Ghio et al., 2014), again compared to SiO_2,_ Middle East sands appear to be more of a nuisance-type dust with relatively low toxicity (Wilfong et al 2011). In the study by Ichinose et al (2008b) that reported greater toxicity of Arizona versus Asian sand dust, the shape (pebble or rock) of the Arizona and Asian particles was similar to each other and whilst the particle size of Arizona sand dust was somewhat larger than Asian sand dust, the concentration of SiO_2_ in Arizona sample was somewhat higher than that in Asian one. It is been well documented that the physical nature of sand dust, including shape (particle versus fibre), size and surface area, plays an important role in its cytotoxcity or inflammatory activity. For example, fibrous TiO_2_ is more cytotoxic to rat alveolar macrophages than spherical TiO_2_ (Hirano et al. 2000) whilst for crystalline SiO_2_, a 1.8 μm size has more effect on inflammatory cell development in the lung tissue and BALF than a smaller size (0.7 μm) (Kajiwara et al. 2007). Comparisons with, and composition analyses of urban PM_2.5_ suggest that the allergic inflammatory responses are greater for microbial element (β-glucan)-rich ASD-PM_2.5_ than for organic chemical-rich U-PM_2.5_ (He et al., 2016a).

Although much of the animal work has focused on relatively short-term exposures and acute effects, in an attempt to replicate Asian dust storm events that can occur 3 or 4 times a month during Spring, several animal studies have employed a ‘sub-acute’ weekly dosing schedule that continued for 4 weeks. Workers have also compared acute versus chronic toxicity following a single dose of heated desert sand dust particles by sacrificing animals at intervals from 24 h up to 4 months post installation (Naota et al., 2013, Shimada et al., 2015). Traits of chronic toxicity included collagen deposition with accompanying granuloma formation, possibly a result of an altered regulation of the extracellular matrix. Evidence also exists that the exacerbated immune responses in airways following a four-time co-exposure to ASD with OVA shifts to a suppressive response following an eight-time co-exposure, and that TGF-β1 is key to such a self-defense reaction by repairing airway injury and weakening eosinophilic inflammation enhanced by ASD at an earlier stage (He et al., 2013b). The exposure approaches used in the experimental animal models are also worthy of some discussion. Although inhalation studies are the ideal experimental approach for assessing the effect of ambient particles, many studies summarized in this review chose the easier and less expensive IT administration, and one that has also been proposed as a reliable route for assessing the pulmonary toxicity of particles in rodents (Warheit et al., 2005, Yokohira et al., 2008). Similar histopathological results have been observed for installation and inhalation (Warheit et al. 2005) and the deposited dose in lung can be more precisely determined with the former. It should be borne in mind however that outcomes are quite different between instillation and inhalation (Sun and Shang 2018). Although instillation via a cannula introduced into the trachea is a straightforward method, it can potentially injure the bronchioles and alveolar walls and cause exposed particles to localize and accumulate in the lung tissues (Muhlfeld et al. 2007).

In summary, the experimental research on desert dust on respiratory endpoints describes mechanistic pathways underlying the pathogenesis of human respiratory disorders. In doing so, they provide support for biological plausibility of epidemiological associations between this particulate air pollutant and events including exacerbation of asthma, hospitalization for respiratory infections and seasonal allergic rhinitis. Insightful findings from the *in vitro* literature indicate that the significant amounts of suspended desert sand dust during storm periods may provide a platform to intermix with chemicals on its surfaces, thereby increasing the bioreactivity of PM_2.5_ during dust storm episodes (Ho et al., 2019), and that mineral dust surface reactions are an unrecognized source of toxic organic chemicals in the atmosphere, enhancing the toxicity of aerosols in urban environments (Kameda et al., 2016). Further investigations to elucidate more detailed associations between chemicals of PM_2.5_ and bioreactivity and identify factors affecting the formation rate of the dust-bound NPAHs, (eg relative humidity, solar radiation intensity) will contribute to a more comprehensive understanding of the complex interactions between urban PM and desert phenomena and subsequent effects of diverse atmospheric environments on human health. As for all natural hazards, desert dust storms are a feature of the landscape that cannot be displaced. Efforts can however be adopted to prepare for dust episodes, thereby ensuring people are out of harm’s way when conditions are threatening to health. Identifying vulnerable communities, such as those living in areas experiencing dust events that alter the composition and toxicity of indigenous urban PM, can help prepare community-level responses, increase the community resilience and improve public health outcomes when episodes arise.

## Funding

This review was funded by the World Health Organisation (2019/977968-0). The review was also part supported by the National Institute for Health Research (NIHR) Health Protection Research Unit in Environmental Exposures and Health, a partnership between Public Health England and Imperial College and the MRC Centre for Environment and Health, which is funded by the Medical Research Council (MR/S0196669/1, 2019–2024). This research was funded in part, by the Wellcome Trust (Grant number 209376/Z/17/Z). For the purpose of Open Access, the author has applied a CC BY public copyright licence to any Author Accepted Manuscript version arising from this submission. Infrastructure support was provided by the NIHR Imperial Biomedical Research Centre (RDR03). The views expressed are those of the author(s) and not necessarily those of the WHO, NIHR, Public Health England or the Department of Health and Social Care.

## CRediT authorship contribution statement

**Julia C. Fussell:** Conceptualization, Writing - original draft. **Frank J. Kelly:** Writing - review & editing.

## Declaration of Competing Interest

The authors declare that they have no known competing financial interests or personal relationships that could have appeared to influence the work reported in this paper.
