# Supplementary material for: Mechanisms underlying the health effects of desert sand dust
Source: Environ Int. 2021 Dec;157:106790. doi: 10.1016/j.envint.2021.106790 (PMC8484861; doi:10.1016/j.envint.2021.106790)
Supplement: Supplementary data 1 [file mmc1.docx]

**Table S1: Summary of the animal studies discussed**

For animal studies having an accompanying *in vitro* component, the latter are summarized in blue italic text.

| *Pulmonary inflammation & injury* | | | | |
| --- | --- | --- | --- | --- |
| **Author** | **Animal model** | **Dust source** | **Exposure conditions: route of administration, dose, dust sample** | **Main findings (exposed versus control)** |
| Lei et al. 2004 | Male pulmonary hypertensive rats (body weight 300-350 g) | Airborne Asian sand dust (AASD) sampled in Chung-Li, Taiwan | Nose-only inhalation of AASD CAPs  315.6 μg/m^3^; 6 h  or  684.5 μg/m^3^; 4.5 h  Rats sacrificed 36 h after exposure | Peripheral blood:  Dose dependent ↑in WBC  ≈ RBCs, haemoglobin, haematocrit, platelets  BALF:  Dose dependent ↑ in total cell count, proportion of neutrophils, total protein, LDH activity, IL-6  ≈ macrophages, lymphocytes, eosinophils |
| Ichinose et al. 2005 | Male ICR mice (5 weeks of age) | ASD collected from Maowusu desert (MFP)  ASD collected from Shapotou (SFP)  AASD sampled in Beijing | IT; 50, 100, 200 μg/animal/wk for 4 weeks  To one of the following samples:  MFP  SFP  SFP+SO_4_  AASD | Lung morphology: all dust particles caused neutrophilic inflammation (greatest for AASD)  BALF:  Dose dependent ↑ in total cells, neutrophils (except for SFP+SO_4_), lymphocytes, eosinophils for all dust particles  ↑ macrophages at all doses (SFP+SO_4_ & AASD)  Order of increase of free cell contents in BALF: SFP < MFP < SFP+SO_4_^2-^ < ADSP  Dose dependent ↑ in IL-1β, IL-12, IFNγ, MCP-1, KC, MIP-1α, (generally at all dose levels for AASD and at 100 & 200 μg for MFP, SFP, SFP+SO_4_); ↑ TNF at all dose levels for AASD, at 200 μg for ΜFP & at 100 & 200 μg for SFP+SO_4_  SFP+SO4 at the 0.1 and 0.2 mg doses, and NAD at all dose  levels exhibited significant increases of TNF-_ .  Correlations of effects with β-glucan & SO_4_^2-^_4_ content of particles  Order of increase of β-glucan content: SFP = SFP + SO_4_^2-^ < MFP < ADSP  Order of increase of SO_4_^2-^ content: SFP < MFP < SFP + SO_4_^2-^ < ADSP |
| Yanagisawa et al 2007 | Male ICR mice (5 weeks of age) | ASD collected from Shapotou | IT; 250 μg/animal; single dose  To either ASD or H-ASD | Lung morphology: ↑neutrophilic inflammation (ASD>H-ASD)  ASD & H-ASD upregulated (>2-fold) 112 & 36 genes respectively  ASD only: ↑ inflammatory-response genes  ASD only: ↑ expression of chemokine (CX-C motif) ligand 1/KC, chemokine (C-X-C motif) ligand 2/MIP-2, chemokine (C-C motif) ligand 3/MIP-1α and chemokine (C-X-C motif) ligand 10/interferon gamma (IFN-γ)-inducible protein-10 |
| He et al 2012b | Male ICR mice (5 weeks of age)  *RAW264.7 cells* | AASD sampled from Iki-island, Japan | IT; 50 or 200 μg/animal, 4 times at 2 week intervals  To one of the following samples:  H-AASD  KP  KP+H-AASD  *H-AASD (30 μg/ml; 3 & 12 h)*  *KP*  *KP+H-AASD* | Lung pathology: H-AASD at both doses exacerbated KP- induced infiltration of neutrophils in bronchiolar/alveolar compartments, goblet cell proliferation in bronchial epithelium & hypertrophy of airway connective tissue  BALF: H-AASD dose dependently enhanced KP induced ↑in neutrophils, IL-1β, IL-6, IL-12, IFN-γ, TNF-α, KC, MCP-1, MIP-1α  *KP+H-AASD: ↑ mRNA expression of IL-1β, IL-6, IFN-β, KC, MCP-1, MIP-1α*  *↑ Protein level of IL-1β, TNF-α, MCP-1; slight ↑* *in TLR2, NALP3, ASC and capase-1* |
| Naota et al 2010 | Male ICR mice (5 or 6 weeks of age) | ASD collected from Tengger desert  AASD sampled from Tottori, Japan | IT; 50, 200, 800, 3000 μg/animal; single dose  To either H-ASD or H-AASD  Animals sacrificed 24h post instillation | H-ASD & H-AASD: Localized accumulation of particles/infiltration of neutrophils and macrophages in bronchioles & alveoli; intensity of inflammation tended to increase with the amount of particles instilled  H-ASD & H-AASD: Cellular degeneration alveolar walls & bronchial epithelium (at 800 & 3000 μg)  Inflammatory cells of lesions: H-ASD ↑ IL-6, TNFα, iNOS, Cu/Zn-SOD (3000 μg)  BALF: H-ASD ↑ total cell numbers (3000 μg), neutrophils and total protein (800 & 3000 μg) |
| Naota et al 2013  Shimada et al 2015 | Male ICR mice (5 or 6 weeks of age) | ASD collected from Tengger desert | IT; 200, 400, 800, 3000 μg H-ASD/animal; single dose  Animals sacrificed 24h, 1 wk, 1,2,3,4 mo post instillation | Acute inflammation observed 24 h subsided at 1 wk & 1 mo; intensity and frequency of the inflammation tended to increase with the amount of the particles instilled  At 2, 3 & 4 months – focal infiltration of lymphocytes & epithelioid macrophages, granuloma formation & collagen deposition (800 & 3000 μg)  Macrophages: +ve immunolabelling for MMP-9 at 24h, 1, 2 mo and for TIMP-1 at 2, 4 mo (3000 μg) |
| Rattanapinyopituk et al 2013 | Male ICR mice (7 weeks of age; body weight 33-36 g) | ASD collected from Tengger desert | IT; 800 μg ASD/animal (single dose) followed 24 h later by 50 nm gold nanoparticles | Acute focal inflammation  ↑ IL-6, TNF-α, Cu/Zn-SOD, iNOS  Alveolar wall destruction  Type 1 epithelial & endothelial cells: ↑ endocytic vesicles within which gold nanoparticles were observed |
| Shimada et al 2018 | Male ICR mice (5 or 6 weeks of age) fed normal diet or low Zn diet  Normal serum Zn: 93.46 µg/dL ± 11.39)  Low serum Zn (< 40 µg/dL ± 8.30) | ASD collected from Tengger desert | IT; 3000 μg ASD-H/animal; single dose  Animals sacrificed 24h, 1 wk, 1,2,3, mo post instillation | Mice with low serum Zn: more prominent & persistent acute & chronic inflammatory changes, larger number of IL-1β-positive macrophages, decreased positive LC-3 staining & dilated lysosomes containing ASD particles in the cytoplasm of macrophages  Mice with normal level of serum Zn: larger number of TNF-positive macrophages |
| Ghio et al 2014 | Mice | Grey (GS) & red sediment (RS) collected from NE Arizona | IT; 100 μg/animal; single dose  To one of the following samples:  Arizona GS  Arizona RS  CB  SiO_2_  NIST 1649 (ambient PM) | Tracheal lavage: ↑ IL-1β, IL-6, TNF-α, MIP-2; ↑ total protein, albumin, NAG, LDH, total cell number &  neutrophils  Order of potency: SiO_2_ & GS & RS > CB & NIST 1649 |
| Wilfong et al 2011 | Male Sprague-Dawley rats (8-9 weeks of age; body weight 226 -275 g | Middle Εast (PM_10_) sand from Camp Buerhing, Kuwait | IT; 1000, 5000 or 10,000 μg/animal; single dose  To one of the following samples:  Middle East PM_10_  SiO_2_  TiO_2_ | SiO_2_:  early, pronounced, sustained inflammation (↑total protein, activities of LDH & β-GLU in BALF) at 5000 & 10,000 μg  TiO_2_ & Middle East PM_10_:  lower magnitude & transient changes in at 5000 & 10,000 μg  Order of potency SiO_2_ > Middle East PM_10_ > TiO_2_ |
| Dorman et al 2012 | Male Sprague-Dawley rats (8 weeks of age) | Iraqi sand (IS) collected from Camp Victory | 6 week nose-only inhalation to air or MSCS (3 h/d, 5d/wk) with IS; 1000 μg /m^3^ 19h/day for last 2 weeks  Groups assigned to following samples:  MSCS  Air+IS  MSCS+IS  Air+SiO_2_  MSCS+SiO_2_ | IS: minimal toxicological response, limited to mild inflammation in the anterior nose & lung,  MSCS +/- co-exposure to IS or SiO_2_: pulmonary inflammation & stress  SiO_2_-MSCS: exacerbation of certain histopathologic responses  IS-MSCS: effects not potentiated |
| Taylor et al 2013 | Male Sprague-Dawley rats (10 weeks of age; body weight 300-320)  *RLE-6TN rat lung epithelial cells* | Sand particles collected from:  Camp Victory, Taji, (Iraq), Khost, (Afghanistan)  *Sand particles collected from:*  *Camp Victory , Taji & Talil (Iraq), Khost (Afghanistan)* | IT; 100 μg/animal; single dose  Camp Victory extract  Taji extract  Khost extract  *250 μg 8- 24 h*  *Camp Victory extract*  *Taji extract*  *Talil extract*  *Khost extract* | Taji extract: acute irritant response  Afghanistan or Taji sand extracts: BALF changes suggestive of mild lung inflammation  Camp Victory extract:  minimal biochemical or cytological BALF changes  Similar lung pathology for all extracts  Not possible to elucidate individual toxic metal(s) that contributed to toxicity  *Relative cytotoxicity (MTT assay, LDH leakage, morphological changes):*  *Taji=Talil>Afghanistan>Camp Victory=Fort Irwin.* |
| *Allergic lung inflammation* | | | | |
| **Author** | **Animal model** | **Dust source** | **Exposure conditions: route of administration, dose, dust sample** | **Main findings (exposed versus control)** |
| Ichinose et al 2008a | Male ICR mice (5 weeks of age)  *RAW264.7 cells* | ASD collected from Shapotou Desert | IT; 100 μg/animal, 4 times at 2 week intervals  To one of the following samples:  ASD  H-ASD  OVA  OVA+ASD  OVA+H-ASD  *ASD (30 μg/ml; 3 h)*  *H-ASD (30 μg/ml; 3 h)* | Lung pathology: ↑ OVA-induced eosinophils in alveoli & airway submucosa (ASD>H-ASD)  BALF: ↑ neutrophils, KC, IL-12, IFNα, RANTES, MIP-α (ASD only except for MIP-α); ↑ OVA-induced eosinophils, IF-5, MCP-3, eotaxin, IgE, IgG1 (ASD>H-ASD)  *ASD: ↑ TLR 2 (not TLR 4) mRNA expression*  *H-ASD: caused no expression of either TLR mRNA* |
| He et al 2010 | Male ICR mice (5 weeks of age)  *RAW264.7 cells* | AASD sampled at Iki-island | IT; 200 μg/animal, 4 times at 2 week intervals  To one of the following samples:  AASD  H-AASD  OVA  OVA+AASD  OVA+H-AASD  *AASD (3 μg/ml; 1 or 3 h)*  *H-AASD (3 μg/ml; 1 or 3 h)* | Lung pathology: ↑ OVA-induced eosinophils in alveoli & airway submucosa  WLLF: ↑ OVA-induced neutrophils, IL-13, IL-5, eotaxin & MCP-3  Serum: ↑ OVA-specific IgE & IgG  Order of the potency: AASD>H-AASD  *AASD (not H-AASD): ↑ expression of TLR 2 (not TLR 4), NALP3, ASC & IL-1β mRNA; ↑ expression IL-1β, IL-6 (not IL-18)* |
| He et al 2013a | Male CD-1 mice (5 weeks of age)  *Cultured BMDMs from WT, TLR2−/−, TLR4−/− & MyD88−/− BALB/c and C57BL/6J mice* | AASD1 originating from Badanjilin desert & sampled from Fukuoka, Japan  AASD2 originating from Hunshandake desert & sampled from Fukuoka, Japan  LPS, β-glucan: AASD1<AASD2) SiO_2_/SO_4_^2-^: AASD1>AASD2) | IT; 100 μg AASD1 or AASD2/animal, 4 times at 2 week intervals  *AASD2 (20 μg/ml; 12 h)* | Lung pathology: ↑ OVA-induced eosinophils in submucosa of the airway & goblet cell proliferation in the bronchial epithelium (AASD2>AASD1)  BALF: ↑ OVA-induced IL-5, IL-13 (ASD1<ASD2) and eotaxin (ASD1>ASD2) in ΒALF  *↑ secretion of IL-6, IL-12, TNF-α, MCP-1 & MIP-1α*  *Cytokine higher in ASD-stimulated TLR2(-/-) cells than in TLR4(-/-) cells, & lower/undetectable in TLR2/4(-/-) & MyD88(-/-) cells* |
| He et al 2016b | WT, TLR4^-/-^, TLR2^-/-^ , MyD88^-/-^ BALB/c background mice (8 weeks of age)  *Cultured BMDMs from WT, TLR2−/−, TLR4−/− & MyD88−/− BALB/c and WT, TLR2−/−, TLR4−/−, TLR2/4-/- & MyD88−/− C57BL/6J mice* | AASD originating from Badanjilin desert & sampled from Fukuoka, Japan | IT; 100 μg/animal; single dose  To one of the following samples:  AASD  OVA  OVA+AASD  *AASD (20 μg/ml; 12 h)* | Lung morphology: ↑ OVA-induced proliferation of goblet cells in airway epithelium & infiltration of eosinophils, neutrophils & lymphocytes into the airway submucosa in WT, TLR2- & TLR4-deficient mice  Serum: ↑ OVA-specific IgE & IgG  Responses similar to WT observed in TLRs 2(-/-), 4(-/-) mice, but not in MyD88(-/-) mice.  *↑ secretion of IL-6, IL-12, TNF-α, MCP-1 & MIP-1α*  *IL-6, IL-12 production in BMDMs was higher in ASD-stimulated TLR2(-/-) cells than in TLR4(-/-) cells, & lower/undetectable in TLR2/4(-/-) and MyD88(-/-) cells* |
| Ren et al 2014b | Male BALB/c mice (6 weeks of age) | ASD collected from Gobi desert | IT; 100 μg/animal, 4 times at 2 week intervals  To one of 12 testing samples containing mixed or individual solutions of LPS (1 or 10 ng), H-ASD & OVA | H-ASD aggravated the LPS-induced (10 mg) neutrophilic lung inflammation along & ↑ IL-12, RANTES, MCP-1, IL-6, and TNF-α  OVA+LPS (1 & 10 ng): ↑ eosinophils slightly & ↑ trace levels of IL-5 & IL-13  OVA+H-ASD+LPS: severe eosinophil infiltration & goblet cell proliferation in airways; remarkable ↑ BALF IL-5 & IL-13; ↑ OVA-specific IgG & IgE in serum  Overall response: LPS 1 ng > LPS 10 ng |
| Ichinose et al 2006 | Male ICR mice (5 weeks of age) | ASD collected from Maowusu desert (SD-1)  ASD collected from Tengger desert (SD-2) | IT; 100 μg/animal, 4 times at 2 week intervals  To one of the following samples:  SD1  SD2  D. farinae  D. farinae+SD1  D. farinae+SD2 | Lung morphology: ↑ D. farina-induced eosinophil airway infiltration & goblet-cell proliferation  BALF: ↑ D. farina-induced IL-5, MCP-1, eotaxin; SD1 ↑ IFN-γ with/without D. farinae |
| Sadakane et al 2016 | Male BALB/c mice (7 weeks of age) | ASD collected from the Gobi desert | IT; 100 μg/animal; single dose  To one of 8 testing samples containing mixed or individual solutions of ZymA, H-ASD & OVA | ZymA: little effect  OVA+H-ASD  ↑ recruitment of inflammatory cells to the lungs; ↑ serum OVA-specific IgE and IgG1  OVA+ZymA+H-ASD  Marked recruitment of eosinophils; ↑ BALF IL-4, IL-13, IL-6, eotaxin/CCL11, MCP-3/CCL7; ↑ serum OVA-specific IgE |
| Sadakane et al 2019 | Male BALB/c mice (7 weeks of age) | ASD collected from the Gobi desert | IT; 100 μg/animal; single dose  To one of 9 testing samples containing mixed or individual solutions of ZymA, LPS, H-ASD & OVA | OVA+LPS  ↑ lung inflammatory cells, particularly neutrophils  + H-ASD potentiated effect  OVA+ZymA  Little effect  + H-ASD stimulated recruitment of eosinophils & serum OVA-specific IgE & IgG1  OVA+LPS+ZymA+H-ASD Affected a few allergic parameters additively or synergistically |
| Liu et al 2014 | Male CD-1 mice (5 weeks of age) | AASD sampled from Fukuoka, Japan  *B. adusta* obtained from AASD aerosol was inactivated by formalin | IT; 100 μg/animal; single dose  To one of 12 testing samples containing mixed or individual solutions of *B. adusta* (2 and 8 μg),  H-AASD & OVA | H-AASD aggravated *B. adusta* induced lung eosinophilia & ↑ BALF inflammatory cell numbers, IL-1β, IL-5, IL-6, IL-12, IL-13, Eotaxin, RANTES, MIP-1α, KC, MPC-1, MCP-3,  *B. adusta* aggravated OVA induced lung eosinophilia  OVA+H-ASD+*B. adusta*: caused most extreme exacerbation to allergic airway inflammation & remarkable ↑ BALF IL-13, eotaxin, IL-5, MCP-3 |
| Ichinose et al 2009 | Male Hartley guinea pigs (5 weeks of age; body weight 290 g) | ASD collected from Shapotou Desert | Nasal inhalation; 300 or 600 μg ASD/animal /wk for 7 weeks plus JCP | ASD ↑ JCP-associated nasal obstructing response but not number of sneezes or nasal secretions  NCLF: ASD ↑ JCP-associated cysteinyl leukotrienes (C_4_, D_4_, E_4_), histamine production & no. eosinophils  ASD ↑ JCP-associated:   - Eosinophil recruitment in nasal mucosa - Goblet cell proliferation in nasal epithelium - Total serum IgE |
| He et al 2014 | Male ICR mice (5 weeks of age)  *BMDM from wild-type, TLR22/2 &*  *TLR42/2 mice* | AASD sampled from Fukuoka,  Japan | IT; 100 μg/animal, 4 times at 2 week intervals  To one of the following samples:  *B. adusta* (2 μg)  *B. adusta* (8 μg)  *B. adusta* (2 μg) + H-AASD  *B. adusta* (8 μg) + H-AASD  *AASD (20 μg/ml; 12 h)*  *B. adusta*  *B. adusta + H-AASD* | H-AASD + *B. adusta*  Lung morphology: lung eosinophilia, proliferation of goblet cells in the airway & fibrous thickening of the subepithelial layer  BALF: ↑ expression of Th2 cytokines & eosinophil-related cytokine & chemokine expression  *Co-exposure: ↑ activation of NF-κB ; ↑ expression of IL-6, MCP-1, IL-12* |
| Hiyoshi et al 2005 | Male ICR mice (5 weeks of age) | ASD collected from Shapotou desert | IT; 100 μg ASD/animal; single dose  To one of the following samples:  ASD  ASD+SO_4_  OVA+ASD  OVA+ASD+SO_4_  Saline | Lung morphology: ↑ neutrophils; ↑ OVA-induced eosinophil recruitment in alveoli & airway  BALF: ↑ OVA-induced IL-5 & MCP-1  No additional effect by SO_4_^2-^ |
| Ichinose et al 2008b | Male ICR mice (5 weeks of age) | ASD collected rom Shapotou Desert  Arizona SD | IT; 100 μg/animal, 4 times at 2 week intervals  To one of the following samples:  H-ASD ± OVA  H-Arizona SD ± OVA  SiO_2_ ± OVA  Al_2_O_3_ ± OVA | Lung pathology: ↑ OVA-induced eosinophils in submucosa of the airway & goblet cell proliferation in the bronchial epithelium.  BALF: ↑ neutrophils, KC, IFN-γ and MIP-α (Arizona SD-H alone); ↑ neutrophils and IL-12, IFN-γ, TNF-α, KC, MIP-1α (SiO_2_ alone); ↑ OVA-induced eosinophils (except Al_2_O_3_), IL-5 & MCP-3 (Arizona SD-H & SiO2)  Overall effects:  OVA+Al_2_O_3_ < OVA+ASD-H < OVA + Arizona SD-H < OVA +SiO_2_ |
| Ren et al 2014a | Male ICR mice (6 weeks of age) | ASD collected from Gobi desert | IT; 100 μg/animal; single dose  To one of 12 testing samples containing mixed or individual solutions of Tar (1 and 5 μg), H-ASD & OVA | H-ASD+OVA: relatively small pathological changes  OVA+ASD+Tar (1 μg): severe eosinophil infiltration & proliferation of goblet cells in the airways; ↑ BALF IL-5 & IL-13 in BALF; adjuvant effect on OVA-specific IgG1 production |
| He et al 2016a | Male BALB/c mice (7 weeks of age) | Urban PM_2.5_ collected during hazy weather in a Shenyang, China  AASD sampled from Fukuoka, Japan | IT; 100 μg/animal, 4 times at 2 week intervals  To one of the following samples:  AASD  U-PM_2.5_  OVA+AASD  OVA+U-PM_2.5_ | Lung pathology: ↑ OVA-induced eosinophils in submucosa of the airway & goblet cell proliferation in the bronchial epithelium  BALF: ↑ OVA-induced eosinophil-relevant cytokines and a chemokine  Serum: ↑ OVA-specific IgG1 and IgE  Lung tissue: ↑ F4/80^+^ CD11b^+^ cells (AASD > U-PM_2.5_) |
| He et al 2019 | Male BALB/c mice (7 weeks of age) | ASD collected from Gobi desert | IT; 100 μg/animal; 4 times at 2 week intervals  To one of 11 testing samples containing mixed or individual solutions of OVA, LPS, urban PM_2.5_, ASD, NAC & DFO | BALF: LPS+PM2.5 & LPS+ASD ↑ OVA-induced eosinophilia, T-helper 2 cytokine & eosinophil-relevant chemokine production  BALF: ↑ OVA-induced eosinophil-relevant cytokines and a chemokine  NAC & DFP findings suggestive that Fe & oxidative stress at least partly involved in enhanced lung eosinophilia caused by LPS with ASD. |
| He et al 2012a | Male CD-1 mice (5 weeks of age) | AASD sampled at Iki-island | IT; 200 μg/animal; single dose  To one of the following samples:  OVA + AASD pre  OVA + AASD sim  OVA + AASD post | Lung pathology: ↑ OVA-induced eosinophils in submucosa of the airway & goblet cell proliferation in the bronchial epithelium.  BALF: ↑ OVA-induced eosinophils, neutrophils, macrophages, lymphocytes,  Th2-associated effecter molecules, eosinophil relevant cytokine & chemokines  Serum: ↑ OVA-specific IgG but not IgE  Order of the potency: OVA+ASD pre < OVA+ASD post < OVA+ASD sim |
| He et al 2013b | Male CD-1 mice (5 weeks of age) | AASD sampled at Iki-island | IT; 100 μg x 4 over 6 weeks or x 8 over 14 weeks  To one of the following samples:  AASD (x 4)  AASD (x 8)  OVA+AASD (x 4)  OVA+AASD (x 8) | OVA+AASD (x 4)  Aggravate allergic airway inflammation; BALF: ↑ IL-13; IL-5, Eotaxin; MCP-3;  fibrous thickening of airway  OVA+AASD (x 8)  Attenuated effects seen with AASD (x 4); BALF: ↑ TGF-β1  Serum ↑ OVA-specific IgE & IgG in x 4 and x 8 exposure |
| Jung et al 2012 | Female BALB/c mice (6 weeks of age)  *Human NCI-H292 cells* | AASD sampled from Incheon City, Korea | Inhalation through a nebulizer; 10,000 μg for 15 min/d for 7 days  AASD  OVA  OVA+AASD  *AASD (10, 100, 250, 500 μg/ml; 24 h)* | Nasal epithelial tissues:  ↑eosinophils & PAS-positive cells  ↑ MUC5AC & TGF-α-immunopositive cells  Order of potency OVA+AAS > OVA or AASD  *↑ MUC5AC- & PAC-immunopositive cells*  *↑ MUC5AC mRNA with increasing AASD concentrations* |
| Kang et al 2012 | Female BALB/c mice (6 weeks of age) | AASD sampled from Incheon City, Korea | Inhalation through a nebulizer; 10,000 μg for 15 min/d for 7 days  AASD  OVA  OVA+AASD | Serum: ↑ IgE  BALF: ↑ IL-4 & IL-5  Lung tissue:  ↑eosinophils & PAS-positive cells  ↑ MUC5AC & TGF-α-immunopositive cells  ↑ IL-4 & IL-5  Order of potency OVA+AAS > OVA or AASD |

| *Systemic toxicity* | | | | |
| --- | --- | --- | --- | --- |
| **Author** | **Animal model** | **Dust source** | **Exposure conditions: route of administration, dose, dust sample** | **Main findings (exposed versus control)** |
| Cao et al 2018 | Male Wistar rats (6 weeks of age; body weight 180 ± 20 g ) | PM collected from Alxa Plateau, Inner Mongolia | Inhalation using a simulated dust storm environment using wind tunnel system; 9000 μg/m^3^ 5h/day for 45, 90, 135, 180 d | Peripheral blood: ↑ WBC & RBC (at d 45 & 180); ↑ IL‑1β, TNF‑α, IL‑6, TGF‑β1; ↓ GSH, SOD; ↑ iNOS  Pathological changes in the lung, kidney & spleen but not heart, liver, stomach, lung & thymus |
| *Lymphoid organs* | | | | |
| **Author** | **Animal model** | **Dust source** | **Exposure conditions: route of administration, dose, dust sample** | **Main findings (exposed versus control)** |
| Song et al 2015 | Male ICR mice (10 weeks of age) | AASD collected from Kitakyushu, Japan | IT; 100 μg AASD/animal | BALF (d1): ↑TNF-α  Splenocytes (d3): ↑ mitogen-induced IL-2, TNF-α, IL-6 production; ↑ activation of NF-κβ CD4^+^ and CD11b^+^ cells |
| Song et al 2019 | WT mice  TLR4^-/-^ mice  TLR2^-/-^ mice  MyD88^-/-^ mice  (All in a BALB/c background;  5 weeks of age) | AASD collected from Kitakyushu, Japan | IT; 100 μg AASD or H-AASD/animal every 2 weeks for 8 weeks | Splenocytes:  ↑ activation in TLR2^−/−^ but not TLR4^−/−^ mice  ↑ concanavalin A‐induced IL-2 production in WT & TLR2^-/-^ but not in TLR4^-/-^ or MyD88^-/-^ mice  NF-κβ activation in WT but not in TLR4^−/−^ or MyD88^−/−^ mice  H-AASD induced p65 phosphorylation in WT & TLR2^−/−^ mice (but not in TLR4^−/−^ & MyD88^−/−^ mice)  CD4^+^, CD11b^+^, CD8^+^, CD45R/B220^+^ cells  H-AASD induced p65 phosphorylation in CD4+ cells (but not in CD8+, CD11b+ & CD45R/B220+ cells) in WT mice  p65 phosphorylation not induced in CD4+, CD8+, CD11b+ or CD45R/B220+ cells post H-AASD administration in TLR4^−/−^ mice |

**Abbreviations**: Al_2_O_3_: aluminum oxide; ASC: apoptosis-associated speck-like protein containing a caspase activating and recruitment domain; ASD: Asian sand dust; AASD: ambient Asian sand dust; BALF: bronchial alveolar lavage fluid; BMDM: bone marrow-derived macrophages; CAPs: concentrated ambient particles; D. farina: Dermatophagoides farina; DFO: deferoxamine; GSH: glutathione; H-AASD: heat treated ambient Asian sand dust; β-GLU: β-glucuronidase; H-ASD: heat treated Asian sand dust; HMEEC: Human middle ear epithelial cells; IFN: interferon; Ig: immunoglobulin; IL: interleukin; IT: intratracheal; iNOS: nitric oxide synthase; JCP: Japanese cedar pollen; KC: keratinocyte chemoattractant; KP: Klebsiella pneumonia; LDH: lactase dehydrogenase; LOAEL: lowest observed adverse effect level; LPS: lipopolysaccharide; MCP: monocyte chemotactic protein; MIP-1α: macrophage inflammatory protein; MMP: matrix metalloproteinase; MPC: mitochondrial pyruvate carrier; MSCS: mainstream cigarette smoke; MyD88: Myeloid differentiation factor 88; NAC: N-acetylcysteine; NAG: N-acetyl-beta-D-glucosaminidase; NALP3: NACHT domain, leucine-rich repeat, and pyrin domain-containing protein 3; NDRA: Nellis Dunes Recreation Area; NF-κB: nuclear factor kappa-light-chain-enhancer of activated B cells: NK: natural killer; NOAEL: no observed adverse effect level; OS: oxidative stress; OVA: ovalbumin; RANTES: regulated on activation normal T expressed and secreted; RBC: red blood cell; SD: sand dust; SiO_2_: silica; SO_4_^2-^: sulphate; SOD: superoxide dismutase; TGF: transforming growth factor; TIMP: tissue inhibitor of metalloproteinase; TiO_2_: titanium dioxide; TLR: toll cell receptor; TNF: tumor necrosis factor; U-PM_2.5_: urban PM_2.5_; WBC: white blood cells; WLLF: whole lung lavage fluid; WT: wild type; Zym: zymosan
