# Supplementary material for: Mechanisms underlying the health effects of desert sand dust
Source: Environ Int. 2021 Dec;157:106790. doi: 10.1016/j.envint.2021.106790 (PMC8484861; doi:10.1016/j.envint.2021.106790)
Supplement: Supplementary data 2 [file mmc2.docx]

**Table S2: Summary of the *in vitro* studies discussed**

| **Author** | ***In vitro* system** | **Dust source** | **Exposure conditions: dose; dust sample treatment groups** | **Main findings** |
| --- | --- | --- | --- | --- |
| **RESPIRATORY AND IMMUNE SYSTEMS** | | | | |
| *Epithelial cell activation & eosinophil migration* | | | | |
| Shin et al 2013 | Cultured human bronchial epithelial cells (BEAS-2B)  Eosinophils isolated from normal human volunteers | ASD collected from Gobi Desert (GB)  AASD sampled from Incheon, Korea (PM_50_)  Smaller H-AASD (PM_10_) | 10-100 μg/ml; 24 & 48 h  Treatment groups:  GB  AASD PM_50_  H-AASD PM_10_ | AASD PM_50_ & PM_10_: ↑ IL-6, IL-8, RANTES production; eosinophil activation & migration  GB: ↑ IL-6  Concentrations & exposure times of the ASDs did not influence the production of IL-6; IL-8 production increased in concentration- and time-dependent manners. RANTES production increased depending on exposure time, but not concentration |
| Honda et al 2014 | Cultured human airway epithelial cells (BEAS-2B cells)  BMDC from atopic prone mice  Splenocytes from atopic prone mice | AASD sampled from Kitakyushu, Japan on 2 different dates (AASD1 & AASD 2) | 3, 30 or 90 μg/ml; 4 or 24 or 72 h  Treatment groups:  AASD1  H-AASD1  AASD2 | AASD1&2: dose dependently ↓ cell viability ; at a dose of 90 μg/ml cell viability was decreased by approximately 10–15%  AASD1&2: dose dependently ↑ expression of IL-6, IL-8, ICAM-1 of BEAS-2B cells and ↑ protein expression of DEC205 in BMDC & BMDC splenocyte proliferation at 72h; most effects at 30 & 90 μg/ml  H-AASD did not change most of these endpoints |
| Honda et al 2017 | Cultured human airway epithelial cells (BEAS-2B cells)  BMDC from atopic prone mice  Splenocytes from atopic prone mice | ASD collected from the Gobi Desert | 50 μg/ml; 24 h  Treatment groups:  H-ASD  *B. adusta*  BaP  H-ASD+ *B. adusta*  H-ASD+ BaP | *B. adusta* & BaP +/- H-ASD: ↑ expression of CD86 & DEC205 on APCs  No remarkable effects on activation of splenocytes or proinflammatory responses in BEAS-2B cells |
| *Mast cell granulation & cytokine release* | | | | |
| Yamada et al 2012 | Rat basophilic leukemia (RBL-2H3) cells | AASD sampled from Naha, Fukuoka & Tsukuba (Japan) | 1/125 – 1/50 dilution; 16 h  Treatment groups:  Naha AASD  Fukuoka AASD  Tsukuba AASD | AASD:  ↑ β-hex release (effect significant at 1/100 dilution)  ↑ TNF-α production (1/100 dilution) |
| *RV infection* | | | | |
| Yeo et al 2010 | Cultured primary human nasal epithelial cells | AASD collected from Gachon University, Korea | 100 μg AASD/ml 24 prior to and 48h post RV infection | AASD: ↑ RV replication; ↑ RV-induced IFN-γ, IL-1β, IL-6, IL-8 mRNA & secretion |
| *Mucus/saliva* | | | | |
| Kim et al 2011 | Cultured nasal polyp epithelial cells | AASD sampled from Incheon, Korea | 0-250 μg AASD/ml; 72 hours | AASD ↑ IL-8 & GM-CSF (not IL-6) ↑ MUC4 and MUC5B mRNA expression  Cytokine expression highest at 100 μg/ml; MUC4 & MUC5B significantly increased at 10 & 50 μg/ml  Insignificant cytotoxic effects |
| Choi et al 2015 | Cultured NCIH292 cells  Primary cultures of normal human nasal epithelial cells | ASD | 10-80 μg ASD/ml | ASD ↑ Expression of MUC8 & MUC5B (attenuated by inhibitors & knockdowns of ERK, MAPK & TLR 4); expressions peaked after 8h of treatment with 40 μg/ml after 10-120 minutes  ASD activated phosphorylation of ERK1/2 & p38 MAPK at 40 μg/ml  ASD ↑ Expression of TLR 4 (not TLR 2) mRNA in a dose-dependent manner (effect significant at 10 μg/ml |
| Penconek et al 2019 | Artificial mucus & saliva models | Arizona transported desert dust (ATDD) | 60 - 6000 μg ATDD /l | ↑ viscosity of saliva & mucus  No effect on diffusion of rhodamine throughout mucus |
| **CYTOTOXICITY &/OR OXIDATIVE STRESS/ NITROSATIVE STRESS** | | | | |
| Kim et al 2003b | Cultured rat alveolar epithelial cells | ASD collected from loess layer in Gunsu Province, China | 100 μg/cm^2^; 18 h  Treatment groups:  ASD  SiO_2_  TiO_2_ | ↓ cell viability  (SiO_2_>ASD>TiO_2_)  ↑ H_2_O_2_  (SiO_2_=ASD=TiO_2_)  ↑ nitrite formation  (SiO_2_>TiO_2_>ASD)  ↑ Fenton activity (ASD>SiO_2_)  ↑ TNF-α (SiO_2_>ASD) |
| Prahalad et al 2011 | Free dG  Calf thymus DNA | Arizona SD | 1000 μg/ml; 15 min  Treatment groups:  Arizona SD  CFA  OFA & ROFA  Ambient PM_2.5_ (SRM 1649 & DUSS) | Particles induced:  8-oxo-dG in calf thymus DNA & dG hydroxylation to 8-oxo-dG in a similar pattern  OFA, ROFA, SRM & DUSS: Elicited significant 3.7, 20.7, 2.1 and 2.4-fold increases.  Arizona SD & ASD:  Elicited slightly elevated but not significant effects |
| Geng et al 2005 & 2006 | Cultures of rat alveolar macrophages | AASD from Wuwei, Gansu Province, China  AASD from Baotou, Inner Mongolia, China | 33, 100 & 300 μg AASD or ambient PM_2.5_ /ml; 4 h | ↓ GSH & ↑ MDA in a dose-dependent manner, significant at 300 μg/ml  ↑ plasma membrane fragility in a dose-dependent manner, significant at 100-300 μg/ml    ↑ intracellular calcium levels in a dose-dependent manner, significant at 100-300 μg/ml, significant decrease > 150 μg/ml  Concentration-dependent decrease in cell viability,  ASSD=PM_2.5_ |
| Ghio et al 2014 | Cultured BEAS-2B cells (normal human bronchial epithelium) | 2 samples of surface sediment from NE Arizona | 100 μg/ml; 24h  Treatment groups:  Arizona SD  CB  SiO_2_  Ambient PM_2.5_ (NIST 1649) | Response to SDs most closely approximated that of SiO_2_  Cytotoxicity  MAP kinase activation & ↑ TNF-α, IFN-γ, IL-1β & IL-6  ↑ cell oxidant generation (Amplex Red fluorescence) & RNA for SOD-1, HO & COX (greatest following exposures to SiO_2_ & SDs) |
| Ho et al 2019 | Cultured human A549 alveolar epithelial cells | ASD collected from Tengger Desert  AASD sampled from Xi’an during 2 different dust storms (DS1 & DS2)  AASD sampled from Beijing during 2 different dust storms (DS1 & DS2) | 50 μg/ml; 16 h  Treatment groups  ASD  Xi’an AASD during DS1  Xi’an AASD during DS2  Beijing AASD during DS1  Beijing AASD DS2  Normal day PM_2.5_  Pollution episode PM_2.5_ | Compared with ASD, pollution episode PM_2.5_ & Xi’an & Beijing DS1:  ↓ cell viability  ↑ LDH  Cell viability & LDH correlated with PM_2.5_ from biomass & industry during dust storms in Xi'an  LDH correlated with PM_2.5_ from vehicles during dust storms in Beijing |

**Abbreviations**: 1-NP: 1-nitropyrene; 8-oxo-dG: 8-Oxo-2'-deoxyguanosine; AASD: ambient Asian sand dust; APC: antigen presenting cell; *B. adusta*: *Bjerkandera adusta*; BaP: benzo[a]pyrene; β-hex: β-hexosaminidase; BMDMs: bone marrow-derived macrophages; CDD: Chinese desert dust; CFA: coal fly ash; CHO: Chinese Hamster Ovary; COX: cyclooxygenase; dG: 2’-deoxyguanosine; DS: dust storm; ERK1/2: extracellular signal-regulated kinase 1/2; GSH: glutathione; H_2_O_2_: hydrogen peroxide; HO: heme oxygenase; ICAM-1: intercellular adhesion molecule-1; IL: interleukin; LDH: lactate dehydrogenase; MAPK: p38 mitogen-activated protein kinase; MCP-1: monocyte chemotactic protein; MDA: malondialdehyde; MIP-1α: macrophage inflammatory protein; MyD88: Myeloid differentiation factor 88; NPAH: nitrated polycyclic aromatic hydrocarbons; OFA: oil fly ash; PM_2.5._: particulate matter < 2.5μm in diameter; Py: pyrene; RANTES: regulated on activation normal T expressed and secreted; ROFA: residual oil fly ash; RV: rhinovirus; SD: sand dust; SiO_2_: silica; SOD: superoxide dismutase; TiO_2_: titanium dioxide; TLR: toll cell receptor; TNF-α : tumor necrosis factor-α
